# The contribution of community transmission to the burden of hospital-associated pathogens: A systematic scoping review of epidemiological models

**DOI:** 10.1016/j.onehlt.2024.100951

**Published:** 2024-12-16

**Authors:** Gary Lin, Suprena Poleon, Alisa Hamilton, Nalini Salvekar, Manuel Jara, Fardad Haghpanah, Cristina Lanzas, Ashley Hazel, Seth Blumberg, Suzanne Lenhart, Alun L. Lloyd, Anil Vullikanti, Eili Klein, For the CDC MInD Healthcare Network

**Affiliations:** a Johns Hopkins University Applied Physics Laboratory, Laurel, MD, USA; b One Health Trust, Washington DC, USA; c The College Preparatory School, Oakland, CA, USA; d Department of Population Health and Pathobiology, North Carolina State University, Raleigh, NC, USA; e Francis I. Proctor Foundation, University of California, San Francisco, CA, USA; f Department of Mathematics, University of Tennessee, Knoxville, TN, USA; g Biomathematics Graduate Program and Department of Mathematics, North Carolina State University, Raleigh, NC, USA; h Department of Computer Science and Biocomplexity Institute and Initiative, University of Virginia, Charlottesville, VA, USA; i Department of Emergency Medicine and Department of Epidemiology, Johns Hopkins University, Baltimore, MD, USA

**Keywords:** Nosocomial infections, Community transmission, Modeling, Community-hospital Interface, Multi-drug resistant organism

## Abstract

Healthcare-associated infections (HAI), particularly those involving multi-drug resistant organisms (MDRO), pose a significant public health threat. Understanding the transmission of these pathogens in short-term acute care hospitals (STACH) is crucial for effective control. Mathematical and computational models play a key role in studying transmission but often overlook the influence of long-term care facilities (LTCFs) and the broader community on transmission. In a systematic scoping review of 4,733 unique studies from 2016 to 2022, we explored the modeling landscape of the hospital-community interface in HAI-causing pathogen transmission. Among the 29 eligible studies, 28 % (*n = 8*) exclusively modeled LTCFs, 45 % (*n = 13*) focused on non-healthcare-related community settings, and 31 % (*n = 9*) considered both settings. Studies emphasizing screening and contact precautions were more likely to include LTCFs but tended to neglect the wider community. This review emphasizes the crucial need for comprehensive modeling that incorporates the community's impact on both clinical and public health outcomes.

## 1 Introduction

Healthcare-associated infections (HAI) present a significant burden on acute care and long-term care settings. In 2015, there were roughly 687,000 HAIs in acute care hospitals in the United States. Each year, about 72,000 hospital patient deaths are attributed to HAIs [1]. The direct medical costs of HAIs in U.S. hospitals amount to $28.4 billion annually [2]. The morbidity and mortality associated with HAIs are predicted to increase dramatically as the threat of antimicrobial resistance (AMR) progresses [3] and Multi-drug resistant organisms (MDRO) become larger drivers of infections. Despite the enormous cost and burden of HAIs, there is still a limited understanding of how transmission of HAI-causing pathogens circulates outside the acute care facilities. Uncertainty around these mechanisms ultimately undermines the effectiveness of infection control and prevention strategies implemented within the healthcare environment to prevent transmission. Patient transfers between short-term acute-care hospitals (STACHs) or admissions and discharges from skilled nursing facilities to the community can greatly influence the incidence of colonized patients entering healthcare facilities [4]. In addition, reservoirs of HAI-causing pathogens outside STACHs can allow for the sustained introduction of pathogens into acute care facilities, leading to an increase in the prevalence of HAIs. Since colonized patients already have a higher risk of developing invasive disease that leads to longer and riskier hospitalizations, the dangers of infection are magnified by MDROs. Furthermore, increases in colonized and infected patients can magnify the likelihood of onward transmission and increase costs for infection control measures within hospitals [5]. Finally, health behaviors in the outpatient setting, such as antibiotic use or hemodialysis, are associated with greater resistance in HAIs for hospital patients [[6], [7], [8], [9], [10]], causing extended hospital stays [11].

One approach to understanding the transmission of HAI-causing pathogens is to utilize computational and mathematical models that integrate dynamics associated with the transmission, whether fomite or direct person-to-person contact. Most epidemiological modeling can be classified as differential equation-based, agent-based (individual-based) (ABM/IBM), or discrete event simulation. In differential equation-based models, populations are typically modeled as having homogeneous mixing with cohesive contact patterns, while ABM/IBM can explicitly model contact patterns with more nuance and complexity. Different mixing assumptions (e.g., homogenous versus heterogeneous) in contact patterns can result in different behaviors in modeled community transmission. Population mixing can be differentiated for various settings based on movement and social behaviors.

**Fig. 1:**
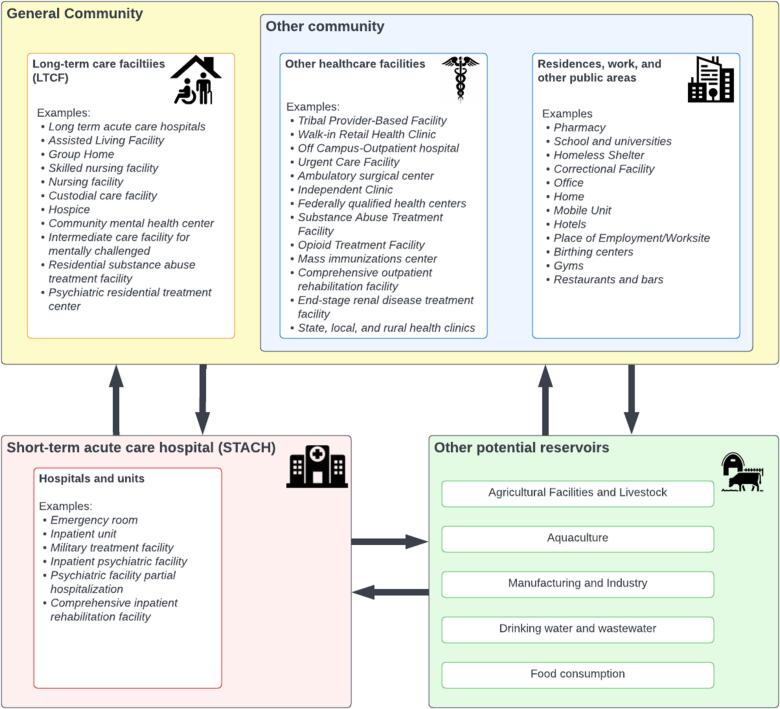
Possible reservoirs and transmission settings for HAI-causing pathogens. In our analysis, we only investigated the general community and STACHs. However, other potential reservoirs of HAI-causing pathogens can exist and contribute to the carriage and transmission of these pathogens.

In this study, we broadly defined *general community* as long-term care facilities (e.g., skilled nursing homes, sub-acute rehabilitation facilities) and other community settings (e.g., residential homes, outpatient offices, ambulatory care centers, hemodialysis centers). Modeling the community-hospital interface can be challenging due to the complex dynamics of healthcare access and transmissions across facilities. Fig. 1 illustrates the delineation of each setting in the One Health context. Previous systematic reviews have suggested that models of *Clostridioides difficile* transmission rarely considered transmission in LTCFs, nursing homes, and communities [12]. Other literature reviews have noted that modeling studies of AMR in LTCFs often lacked movement dynamics between LTCFs and acute-care hospitals [13]. Staff and visitors can also be sources of contamination and HAIs, but are not routinely modeled. [14] Finally, health equity concerns, both locally and globally, necessitate modeling the community to (a) evaluate how dynamics may differ in at-risk populations and (b) the different dynamics that characterize low and middle-income country settings, which are underrepresented in the modeling literature [15].

We employed a systematic scoping review methodology based on PRISMA-ScR guidelines to explore the progress and gaps in mathematical modeling in healthcare epidemiology, specifically the role of community transmission. We highlighted the settings and pathogens, interventions (counterfactuals), model structure and assumptions, population characteristics, movement and transmission characteristics, and the role of data. Subsequently, we investigated the gaps and progress in these studies. Finally, we discussed and provided suggestions to advance the field of infectious disease modeling of HAI-causing pathogens.

## 2 Methods

We investigated modeling studies that were published between January 1, 2016, and June 15, 2022. The studies must include STACHs and the role of transmission of HAI-causing pathogens in community settings outside the STACHs. The following sections outline the search strategy, screening, and full-text review methods utilized for the current study.

### 2.1 Search strategy

The initial search was conducted on four databases: PubMed, Medline, Scopus, and Embase. We developed a comprehensive search query using key terms and controlled vocabulary. We first developed a system of search terms from four broad categories: (1) HAI-causing pathogens, (2) healthcare facility setting, (3) community setting, and (4) modeling methods (see **Fig. S1** in **Appendix A**). For each category, subcategories were derived with search terms used to query each database. Search terms within subcategories were combined using the “OR” Boolean operator, and subcategories were combined using “OR” and “AND” operators in the final search strategy below. The exhaustive list of search terms is presented in **Appendix A**.

### 2.2 Selection criteria

All literature was collected and compiled into the Covidence Platform [16]. We followed the guidelines laid out by PRISMA for scoping reviews [17]. Once the literature was collected from all four databases (Embase, PubMed, Medline, and Scopus), five reviewers conducted the preliminary title and abstract screening on Covidence (S.P., G.L., M.J., N.S., and F.H.). The screening protocol that reviewers used is described in detail in **Appendix B**.

The second phase encompassed reading the full text and determining the eligibility of each study. We compiled the screened studies into a Google Sheet shared among ten reviewers (G.L., S.P., M.L., A.Haz., N.S., A.Ham., S.L., C.L., A.L.L., F.H., and E.K.). Each reviewer read and determined whether the eligibility criteria were met based on the inclusion and exclusion criteria mentioned in the following sections. Afterward, two reviewers (S.P. and G.L.) reassessed each review for quality and accuracy. The questions used to guide the full-text analysis are located in **Appendix C.**

#### 2.2.1 Inclusion criteria

We included studies published as journal articles (e.g., original research or letters). Regarding study design, studies needed to include STACHs (e.g., academic hospitals or ICUs) in their model. Additionally, included studies must incorporate some community settings. We generally define *general community* as long-term care facilities (e.g., nursing homes, long-term acute care facilities) and non-healthcare settings (e.g., households, schools, offices). The intention was to include models that have an interface between the hospital and community where infected patients in the community and hospitals are represented explicitly.

#### 2.2.2 Exclusion criteria

In our preliminary screening and full-text review, systematic reviews, conference presentations, or conference abstracts were excluded from our analysis. We excluded any study that was not in English. Studies that included only admission and discharge rates as a surrogate for community importation were excluded. We also excluded models that looked at community transmission without modeling STACHs explicitly. Studies that relied on machine learning and statistical models were excluded as well.

### 2.3 Data extraction and analysis

Extracted data included the pathogen type, study setting, model structure, and role of data in the modeling process. Ten reviewers (G.L., S.P., M.J., A.Haz., N.S., A.Ham., S.L., C.L., A.L.L., F.H., and E.K.) reviewed each study in detail and answered questions related to the characteristics of each study. The review responses were then compiled into a Microsoft Excel Sheet where studies were tabulated and counted.

## 3 Results

**Fig. 2:**
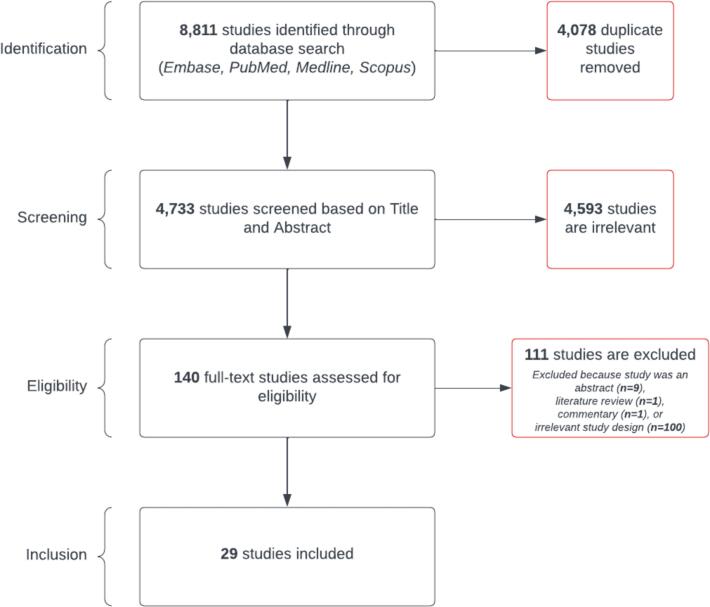
The PRISMA-ScR flowchart showing the inclusion process of the systematic scoping review. During the eligibility stage, exclusion based on “hospital interface” indicates studies that did not have mechanistic relationships or flows between community (homes, nursing homes, long-term care facilities) and acute care hospitals.

Among the 4,733 unique studies identified in the four databases, 29 studies met eligibility criteria for full-text analysis of modeling progress, trends, and gaps, including short-form questions regarding the model structure and study characteristics. A summary of the select study features are in Table 1. The results are comprehensively listed in **Tables S1 - S9** in **Appendix D.** Fig. 2 illustrates the scoping review process and the inclusion and exclusion of studies based on PRISMA-ScR guidelines. Of the 141 studies that met the criteria in the title and abstract screening, 111 studies were excluded based on the selection criteria. The most common reason was “irrelevant study design,” for which 100 studies were excluded. Among the excluded irrelevant study design studies, 16 studies lacked STACHs, 81 studies either lacked community or long-term health facilities in their models, and 45 studies only modeled admission and discharge as a simple fixed importation/exportation rate. Additionally, 11 studies were excluded because they were commentary, literature reviews, or conference abstracts.

**Table 1:** Brief summary of the eligible studies for full-text review.

**Study [Refs]**	**Pathogens**	**Publication Year**	**Settings**	**Model Type**	**Hospital-community interactions**	**Contact Network or Movement**	**Country**
Durham et al. [40]	C.d*iff*	2016	LTCF + Other Community	Stochastic Sim/Gillespie Algorithm	Admission and discharge	No	USA
McLure et al. [18]	C.d*iff*	2019	Other Community	ABM/IBM	Admission and discharge	No	USA
McLure et al. [19]	C.d*iff*	2019	Other Community	ABM/IBM	Admission and discharge	No	USA
Rhea et al. [20]	C.d*iff*	2019	LTCF + Other Community	ABM/IBM	Admission, discharge, transfer, and readmission	Yes	USA
Rhea et al. [21]	C.d*iff*	2020	LTCF	ABM/IBM	Admission, discharge, transfer, and readmission	Yes	USA
Toth et al. [22]	C.d*iff*	2020	LTCF + Other Community	ABM/IBM	Admission, discharge, transfer, and readmission	No	USA
Van Kleef et al. [23]	C.d*iff*	2016	LTCF + Other Community	ABM/IBM	Admission, discharge, and readmission	No	UK
Changruenngam et al. [33]	Carbapenem-resistant *Klebsiella pneumonia*	2022	Other Community	Differential Equations	Admission and discharge	No	Not specified
Bartsch et al. [30]	CRE	2020	LTCF	ABM/IBM	Admission, discharge, transfer, and readmission	Yes	USA
Lee et al. [25]	CRE	2020	LTCF + Other Community	ABM/IBM	Admission, discharge, transfer, and readmission	No	USA
Lee et al. [27]	CRE	2021	LTCF	ABM/IBM	Admission, discharge, transfer, and readmission	Yes	USA
Lee et al. [26]	CRE	2021	LTCF	ABM/IBM	Admission, discharge, transfer, and readmission	Yes	USA
Lee et al. [28]	CRE	2016	LTCF	ABM/IBM	Admission, discharge, transfer, and readmission	Yes	USA
Lin et al. [34]	CRE	2021	LTCF + Other Community	Differential Equations	Admission, discharge, and transfer	Yes	USA
Toth et al. [29]	CRE	2017	LTCF	ABM/IBM	Admission, discharge, transfer, and readmission	Yes	USA
Knight et al. [35]	*E.coli*	2018	Other Community	Differential Equations	Admission and discharge	No	UK
MacFadden et al. [36]	*E.coli*	2019	Other Community	Differential Equations	Admission and discharge	No	Sweden
Talaminos et al. [42]	*E.coli*	2016	LTCF + Other Community	Discrete Event/Microsim	Admission and discharge	No	Spain
Godijk et al. [37]	ESBL-producing *Enterobacteriaceae*	2022	Other Community	Differential Equations	Admission, discharge, and readmission	No	Netherlands
Haverkate et al. [43]	ESBL-producing *Enterobacteriaceae*	2017	Other Community	Markov Model	Admission and discharge	No	Netherlands
Salazar-Vizcaya et al. [41]	ESBL-producing *Klebsiella pneumoniae*	2022	Other Community	Differential Equations	Admission, discharge, transfer, and readmission	No	Switzerland
Bartsch et al. [24]	Generic nosocomial bacteria	2021	LTCF	ABM/IBM	Admission and discharge	Yes	USA
Belik et al. [31]	Generic nosocomial bacteria	2016	Other Community	ABM/IBM	Admission, discharge, and transfer	Yes	Germany
Van Den Dool et al. [46]	Generic nosocomial bacteria	2016	LTCF + Other Community	Discrete Event/Microsim	Admission and discharge	Yes	Netherlands
Van Kleef et al. [38]	Generic nosocomial bacteria	2017	Other Community	Differential Equations	Admission and discharge	No	EU
Di Ruscio et al. [32]	MRSA	2019	LTCF + Other Community	ABM/IBM	Admission and discharge	Yes	Norway
Gowler et al. [39]	MRSA	2022	Other Community	Differential Equations	Admission and discharge	No	Not specified
Rocha et al. [44]	MRSA	2020	LTCF	Network Sim	Admission, discharge, and readmission	Yes	Sweden
Piotrowska et al. [45]	Multidrug resistant *Enterobacteriaceae*	2020	Other Community	Network Sim	Admission, discharge, transfer, and readmission	Yes	Germany

**Key Abbreviations:** ABM = agent-based model; C.Diff = Clostridioides difficile**;** CRE = Carbapenem-resistant Enterobacterales; ESBL = Extended-spectrum β-lactamase; IBM = individual-based model; LTCF = long term care facility; MRSA = Methicillin-resistant *Staphylococcus aureus*.

### 3.1 Model structure and assumptions

**Fig. 3:**
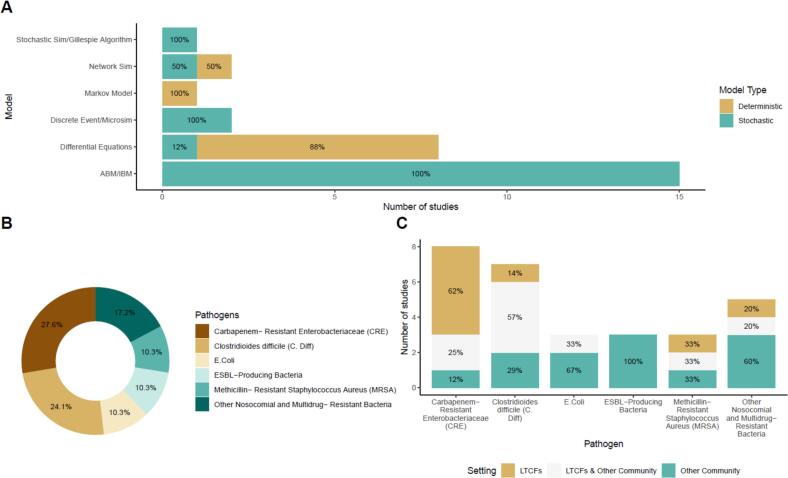
(*A*) Stacked bar chart showing the number of studies with corresponding model types stratified by deterministic and stochastic modeling implementations. (*B*) Pie chart showing the distribution of studies for each class of HAI-causing pathogens modeled for all 29 included studies. (*C*) Stacked bar chart showing the number of studies by modeled settings by pathogens. The plot shows the number of studies for each pathogen and is stacked by modeled settings: long-term care facilities (LTCFs), other community settings, or both (LTCFs & Other Community).

The most common type of model was stochastic agent/individual-based (*n* = 15, 51 %; Fig. 3A) [[18], [19], [20], [21], [22], [23], [24], [25], [26], [27], [28], [29], [30], [31], [32]]. There were eight differential equation-based models (27 %) [23,[33], [34], [35], [36], [37], [38], [39]], and all but one were deterministic [35]. In studies that reported their software implementation, most models were programmed using C++ (*n* = 7, 24 %) [[24], [25], [26], [27], [28],^30^,^40^], followed by R (*n* = 5, 17 %) [35,36,38,39,41]. Among the seven studies programmed in C++, six studies [[24], [25], [26], [27], [28],^30^] utilized the same model.

As with most epidemiological models, individuals' health/disease states were characterized as compartments (e.g., susceptible, infected, and colonized). Most studies included at least three disease states, including differing levels of susceptibility (e.g., high susceptibility due to antimicrobial exposure), infectiousness, or strains (antimicrobial resistant versus antimicrobial susceptible). In our analysis, 13 studies (45 %) [[18], [19], [20],^22^,^23^,^29^,^34^,^36^,^37^,[40], [41], [42], [43]] had varying levels of susceptibility, 11 (38 %) [18,19,22,25,33,35,37,[39], [40], [41],^43^] had varying levels of infectiousness in the infected compartment state, and 4 studies (14 %) [[35], [36], [37], [38]] had multiple pathogenic strains included in their model. We also found that 13 studies (45 %) [22,23,[25], [26], [27], [28], [29], [30],^33^,^34^,^39^,^44^,^45^] included the detection status of colonization.

### 3.2 Settings and pathogens

Table 1 summarizes the studies that were included in our analysis. In the included studies, we found that 9 studies (31 %) included both long-term care settings and other types of community settings (e.g., households and workplaces) in their model [20,22,23,25,32,34,40,42,46], while 8 studies (27 %) only included long-term care settings [21,24,[26], [27], [28], [29], [30],^44^] and 12 studies (41 %) only included other types of community settings [18,19,31,33,[35], [36], [37], [38], [39],^41^,^43^,^45^]. Fig. 3B lists the HAI-causing pathogens that were included in models reviewed; the two most common pathogens modeled were *Clostridioides difficile* (*C. diff*) (*n* = 7, 24 %) [[18], [19], [20], [21], [22], [23],^40^] and Carbapenem-resistant *Enterobacteriaceae* (CRE) (*n* = 7, 24 %) [[25], [26], [27], [28], [29], [30],^34^]. Fig. 3C shows the distribution of transmission settings modeled by the pathogen category. Studies that modeled CRE were more likely to include LTCFs, while studies that modeled *C.diff* included more community settings outside of LTCFs. Most studies utilized data from the United States (*n* = 13, 45 %) [[18], [19], [20], [21], [22],[24], [25], [26], [27], [28], [29], [30],^34^,^40^]. No studies included data from low- and middle-income countries (LMIC).

### 3.3 Study interventions

**Fig. 4:**
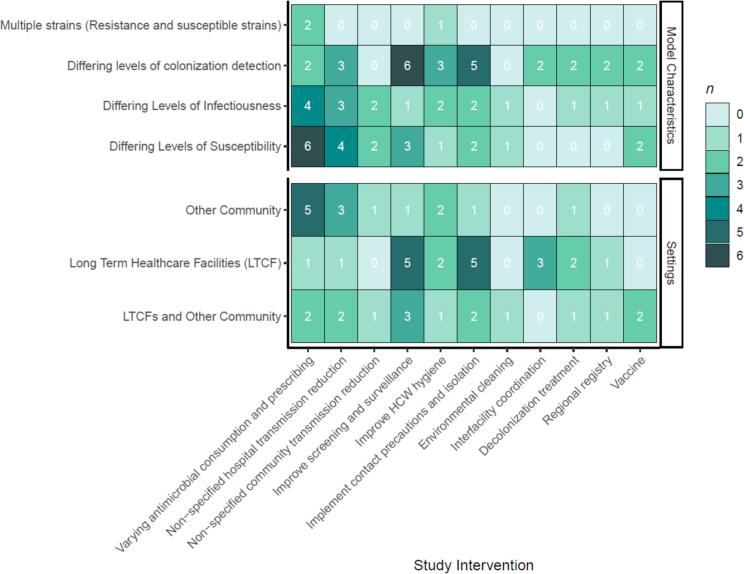
A heatmap showing the co-occurrence of study settings and various model characteristics with different modeled interventions. The number of studies that fit the description is indicated both numerically in the boxes and by shading of the boxes.

Fig. 4 summarizes the reviewed studies by model characteristics and setting. Many studies tested different aspects of an infection, prevention, and control (IPC) measures. Eight studies investigated the implementation of contact precautions and isolation [24,26,[28], [29], [30],^32^,^33^,^40^], and three studies with those interventions included other community settings [32,33,40]. Other IPC interventions include improving HCW hygiene (*n* = 5, 17 %) [26,[38], [39], [40],^44^], environmental cleaning (*n* = 1, 3 %) [40], and decolonization treatment (*n* = 4, 14 %) [24,26,32,39]. Studies also included non-specific abstraction of reduction in hospital transmission (*n* = 6, 21 %) [19,27,34,39,41,42] and community transmission (*n* = 2, 7 %) [19,40].

Studies that investigated improved surveillance and screening (*n* = 9, 31 %) [26,[28], [29], [30], [31], [32],^34^,^40^,^44^] were more likely to include LTCFs (*n* = 8, 28 %) [26,[28], [29], [30],^32^,^34^,^40^,^44^] and less likely to include other community settings (*n* = 4, 14 %) [31,32,34,40]. Three studies (10 %) included both community and LTCF settings [32,34,40]. Studies that included interfacility coordination (*n* = 3, 10 %) [24,28,30] or regional registries (*n* = 2, 7 %) [25,27] interventions where multiple stakeholders were involved usually included LTCFs, but only one study included other community settings [25].

As for pharmaceutical interventions, eight studies that varied antimicrobial consumption and prescribing, i.e., antimicrobial stewardship efforts (*n* = 8, 28 %) [19,21,23,33,36,37,41,42] while two studies investigated the impact of vaccination (*n* = 2, 7 %) [22,23].

### 3.4 Population characteristics

Besides disease states, most models assumed the population was homogenous and that behavior, susceptibility, and transmissibility were identical across all demographic segments of the population. However, six studies (21 %) included age differences in the population [20,21,32,39,40,43], three studies (10 %) included gender in their analysis [20,21,43], two studies (7 %) included race [20,21], and one study (3 %) included ethnicity (immigrant versus non-immigrant) [32]. No study specifically analyzed health disparities between populations beyond reporting population-specific outcomes.

### 3.5 Movement and transmission characteristics

In terms of movement of patients, all models included admissions and discharges to STACHs and/or LTCFs. Readmission was included explicitly in 13 studies (44 %) [[20], [21], [22], [23], [24], [25], [26], [27], [28], [29], [30],^37^,^45^]. There were 12 studies (41 %) that included transfers between STACHs and other healthcare facilities [[20], [21], [22],[24], [25], [26], [27], [28], [29], [30],^34^,^46^]. Among those studies, nine studies (31 %) incorporated transfers between STACHs [[20], [21], [22],^24^,^26^,^27^,^30^,^34^,^44^] and three of them modeled additional movement between STACHs and communities [20,21,34]. One study (3 %) simulated international travel outside the country of interest [32]. Seven studies with spatially defined locations included geospatial features and considerations, including hospital or community settings (*n* = 5, 17 %) [20,21,34,44,45] and movement assumptions based on near proximity to hospitals (*n* = 2, 7 %) [32,46]. We found that two studies (7 %) utilized data to investigate the impacts of movement on colonization in the community setting between high and low-prevalence geographical regions [41,43].

Most studies (*n* = 22, 76 %) assumed direct transmission and did not distinguish between patients and healthcare workers [18,19,22,23,[25], [26], [27], [28], [29],[31], [32], [33], [34], [35], [36],[40], [41], [42], [43], [44], [45], [46]]. Three studies (10 %) that considered HCW-mediated transmission in their analysis, where HCWs explicitly acted as vectors between patients [24,30,38]. Haverkate et al. [43] included hospital-visitor interactions in their model. In three studies (10 %) with healthcare workers, Di Ruscio et al. [32] and van Kleef et al. [38] modeled HCWs identically to the general population (homogenous mixing), while Changruenngam et al. [33] modeled transmission as only possible through HCW-mediated contact networks. Two studies (7 %) with community transmission included zoonotic or foodborne transmission [18,19].

### 3.6 Role of data and parameterization

Among the 29 included studies, all used data to inform their parameters in some manner. There were 14 studies (48 %) [20,21,24,[26], [27], [28], [29], [30], [31], [32],^34^,[43], [44], [45], [46]] that incorporated contact or movement networks, for example transfers between healthcare facilities and contact rate matrix between populations. Parameters in all but two studies [33,42] were informed by primary source data, such as a survey or electronic health records (EHR) data (e.g., admission rates, average length-of-stay), while 16 studies (55 %) were parameterized by fitting their models to data, such as observed cases [[18], [19], [20], [21], [22], [23],^29^,^32^,^33^,^35^,^36^,[39], [40], [41], [42], [43]]. In addition, sensitivity analyses (Latin-hypercube parameter sampling) were performed in 22 studies (76 %) [18,19,[22], [23], [24], [25], [26], [27], [28], [29], [30],^32^,[34], [35], [36],[38], [39], [40], [41], [42], [43],^46^]. Finally, for parameters that could not be directly informed, most studies utilized parameter values from other literature (*n* = 28, 97 %) with Belik et al. being the exception [31]. Most studies (*n* = 23, 79 %) inferred their model parameters based on expert opinion except for six studies [19,31,32,37,44,45]. Only two studies (7 %) quantified the uncertainty of less-known parameters [39,40].

## 4 Discussion

Although there has been progress in incorporating community and LTCF settings in simulating nosocomial infections, most HAI modeling studies still limit themselves by investigating only the healthcare settings and do not include community or long-term care facilities with higher risks of HAIs. Only 29 studies (21 %) out of the 140 studies that were eligible for full-text screening included community settings. While these computational and mathematical models of the hospital and community interface can provide valuable insights into the transmission of HAI-causing pathogens and incidences of infections, there are also limitations to those models to consider.

Overall, the included studies had models tailored to the settings of higher transmission risk. For example, *C. diff* and *E.Coli,* which have a higher risk of transmission in community settings outside of LTCFs, were more likely to include those settings in their model, while CRE, which is primarily nosocomial, was more likely only to include LTCFs (Fig. 3C). However, transmission of these pathogens can be complex and include a myriad of pathways and interactions in healthcare and community environments, making it challenging to capture all mechanisms in a model.

All studies included patient admission and discharges to STACHs and LTCFs. A handful of studies explicitly included movement between healthcare settings, but only a few studies included travel between communities and hospitals or readmission. Some models were more nuanced with patient transfer or movement networks, and those studies were typically stochastic ABMs and included real-world data (e.g., EHRs) to build a patient flow network. In network modeling studies with both communities and LTCFs, agents in communities are characterized by a synthetic population that defines transmission risks based on contacts from stochastic movement in the community [20,32], random mixing [34], or no possibility of transmission [46]. No study leveraged a synthetic population to produce more realistic contact networks.

Model development and validation with empirical data or expert opinion are necessary to assume the generalizability of results. All studies included data to inform the model structure or parameters. A major limitation is the lack of data on transmission and prevalence in the community. Based on the analysis of the role of data and parameterization, roughly half of the studies included information about the contact or movement network, which would introduce added realism to the propagation of colonization events in community settings. Even with a synthetic population, studies with contact networks still utilize random contacts. A small number of those studies conducted uncertainty quantification. Leveraging uncertainty quantification methodologies can provide insights into model behavior where parameters that drive transmission are yet to be quantified. This suggests that most studies that do consider community settings may oversimplify transmission dynamics that impact changes in the importation of HAI-causing pathogens into the healthcare setting.

Models are often used to measure intervention effectiveness before real-world implementation. Studies that include antimicrobial stewardship interventions that vary AMR use focused on other community settings outside of LTCFs, which is typical for those types of programs because AMR-based interventions occur within and outside of healthcare settings. As expected, those studies also include differing levels of susceptibility based on AMR consumption rate, which will alter transmissibility risks in the hospital and community. Durham et al. found that the effect of antimicrobial drug use exacerbated incidence in the community, which is amplified in a high-transmission setting like LTCFs and STACHs [40]. In contrast, we found that most models that included screening and surveillance interventions typically focused on LTCFs and were less likely to include other community settings (Fig. 4). One limitation of testing surveillance of healthcare settings in models without communities is an inaccurate assessment of public health impacts.

### 4.1 Challenges and gaps in modeling transmission of HAI-causing pathogens

Studying the impacts of healthcare-level interventions can be difficult if the community is not sufficiently modeled. Colonization of most HAI bacteria, like *Staphylococcus aureus* and Carbapenem-resistant *Enterobacteriaceae,* tends to be subclinical or asymptomatic, which creates additional difficulties in identifying the importation of carriers from the community upon admission. Identification of colonized patients through surveillance screening can reduce onward transmission when patients are placed under precautions; however, the greater pressure from more colonized patients entering the facility will typically lead to increased nosocomial transmission. In addition to patients themselves, visitors and healthcare workers may import HAI-causing pathogens after acquisition from regular contact with the broader community, including household members, random contacts, and animals. [47]

Similarly, population characteristics such as race, ethnicity, age, and geographic attributes must be accounted for in the model to assess the potential impact of health disparities on both transmission and effectiveness of interventions. None of the identified studies included any health disparity research in their analysis, which can be detrimental to understanding the increased morbidities associated with HAI-causing pathogens in impoverished communities and safety-net hospitals. [48] Additionally, examining the impact of HAIs in vulnerable populations and incorporating population structure, including spatial and age characteristics, can provide valuable insight into the spread of disease. [49]

From a high-level disease importation perspective, no studies were conducted in low or middle-income countries where HAI prevalence is much higher than in Europe and the U.S. [50] Furthermore, most studies in this scoping review did not include international travel, which has been shown to introduce HAI-causing pathogens and antimicrobial-resistant bacteria through importation. [51]

### 4.2 Opportunities and the one health perspective

Understanding the fluctuating and changing prevalence of HAI-causing pathogens can help determine the disease burden on hospitals and other healthcare facilities. Seasonal changes in transmission between seasons can be modeled, given their seasonal variations of HAI-related hospitalization [52]. Given the advances in other infectious disease modeling, we acknowledge tradeoffs in data availability on community prevalence, biological understanding, and research efforts. Transmission models of other pathogens, especially ones that cause upper respiratory infections and circulate widely in the community, such as influenza [53], pertussis [54], MDR-tuberculosis [55], and, more recently, SARS-CoV-2 [56,57], have included the community-hospital interface due to their importance in the disease dynamics and their contributions to nosocomial infections. Household transmissions can be modeled when epidemiological data becomes available, which was the case for SARS-CoV-2 [45]. Spatial features of disease spread have also been modeled [58]. For other infectious diseases, we see advances in understanding human and social behavior, such as HIV [59], where drug use and sexual activity are modeled. Multiscale interactions such as within-host (immune-viral interactions) and between-host (transmission) dynamics have also been investigated for many diseases [60,61].

A One Health approach, which considers the interdependencies of human, animal, and environmentalhealth, can help bridge these gaps and provide a more comprehensive understanding of the role of community reservoirs in HAI incidences. By understanding the gaps in modeling in the One Health context, we can start speculating about the missing information and knowledge, such as data and modeling methods, and begin formalizing a plan to refocus data collection and propose better paradigms in modeling. Given the One Health context, we also need to understand how other reservoirs, shown in Fig. 1, play a role in importing HAI-causing pathogens into the hospital. Understanding multiscale interactions (within-host and between-host) can also better inform our understanding of colonization dynamics in individuals and their contribution to community prevalence. Animal carriage can also play a large role in propagating these pathogens, whether foodborne or zoonotic, and was included in only one study in our review [18].

Community transmission has been extrapolated from admission and discharge rates in many modeling studies investigating HAI-causing pathogens. Although simplifying importation may be sufficient in some studies, understanding the dynamic changes in HAI-causing pathogen prevalence in the community can better inform policies and interventions that reduce HAI prevalence in the hospital. Possible population-level interventions include vaccines and outpatient decolonization. These interventions will impact general communities with no specific place or settings, which were often abstracted in models with no LTCFs [18,19,33,[35], [36], [37], [38], [39],^41^,^45^].

### 4.3 Need for digital and public health surveillance

Most studies investigated STACHs and LTCFs with greater granularity and communities with less granularity. This leads to potential gaps in understanding finer and more nuanced transmission pathways that could occur in non-healthcare settings. Improvements in digital and community surveillance can add granularity to models of the community. Digital surveillance can help construct a synthetic social mobility network that drives the true prevalence of HAI-causing pathogens in the community. Tracking patient movement between hospitals and communities, whether in their homes or a long-term care facilities, while collecting their data either passively (e.g., wastewater monitoring, digital surveillance) or actively (e.g., follow-up sampling) can help models achieve higher fidelity and incorporate more realistic disease dynamics of AMR and HAIs. For example, infection with C. *diff* increases the risk of infection in household members [62]. Even hospitalization without C. *diff* increased the risk of other family members having similar infections [63]. The latter suggests that asymptomatic colonization may play an important role in community and subsequent hospital transmission.

Although studies included in this scoping review capture the community abstractly, further data collection is still needed, whether through active surveillance via sampling or tracking colonized patients through the community. Tracking HAI-causing pathogens in animal populations can inform potential zoonotic and foodborne transmission [47]. McLure et al. [18] modeled non-human populations, such as livestock, which would have benefited from tracking animal populations. Multiple data sources, like wastewater surveillance and EHR data, must be integrated to understand the burden of HAI-causing pathogens fully. Three studies attempted to model households [31,42,43], where community-based surveillance, such as wastewater monitoring, could shed insight into inter-household transmission. Additionally, understanding and modeling health disparities between demographic and social groups may be better informed through enhanced public health surveillance in low-income and underserved areas. Opportunities to improve disease detection in lower and middle-income countries can be achieved through syndromic surveillance and accessible home testing [64].

### 4.4 Review scope and limitations

As with most scoping reviews, this review was subject to some limitations in scope. The review investigated four databases (PubMed, Medline, Scopus, and Embase) using comprehensive search terms but may have missed studies that did not specify their use in modeling. Any study outside of these databases was not included. Additionally, we investigated recent trends and did not include studies before January 1, 2016. Studies that relied on statistical and machine learning models were not selected due to their lack of mechanistic dynamics (i.e., black box). However, these studies may yield additional insights into community transmission that are not mechanistically understood and detect possible causation.

## 5 Conclusion

Computational and mathematical modeling is essential in understanding the transmission of healthcare-associated infections (HAI) pathogens. However, it is vital to recognize the limitations of these models, including dependence on assumptions and input data, difficulty in fully capturing all relevant factors, lack of community-level data, and neglect of health disparities. Accurate assessments of clinical and public health interventions, such as improved screening and contact precautions, require studies to model the disease dynamics in the community. Moreover, modeling communities allow studies to recognize the contributions of external reservoirs on nosocomial transmission and clinical disease prevalence. We propose a One Health approach to identify and bridge these gaps in HAI modeling.

## Authors contribution

G.L. and E.K., were responsible for the study conception and design; G.L. and A.Ham. developed the literature search strategy; S.P., G.L., M.J., N.S., and F.H. conducted the abstract screening; G.L., S.P., M.J., A.Haz., N.S., A.Ham., S.L., C.L., A.L.L., F.H., and E.K. conducted the full-text review; G.L. and S.P. analyzed and interpreted the data; G.L. drafted the manuscript; E.K., S.P., M.J., A.Haz., N.S., A.Ham., S.L., C.L., A.L.L., F.H., and, A.V. reviewed the draft and provided critical feedback; All authors contributed to and approved the final manuscript.

## CRediT authorship contribution statement

**Gary Lin:** Writing – review & editing, Writing – original draft, Visualization, Validation, Supervision, Methodology, Investigation, Formal analysis, Data curation, Conceptualization. **Suprena Poleon:** Writing – review & editing, Formal analysis, Data curation. **Alisa Hamilton:** Writing – review & editing, Methodology, Investigation. **Nalini Salvekar:** Writing – review & editing, Investigation. **Manuel Jara:** Writing – review & editing, Investigation. **Fardad Haghpanah:** Writing – review & editing, Investigation. **Cristina Lanzas:** Writing – review & editing, Investigation. **Ashley Hazel:** Writing – review & editing, Investigation. **Seth Blumberg:** Writing – review & editing, Investigation. **Suzanne Lenhart:** Writing – review & editing, Investigation. **Alun L. Lloyd:** Writing – review & editing, Investigation. **Anil Vullikanti:** Writing – review & editing, Funding acquisition. **Eili Klein:** Writing – review & editing, Investigation, Funding acquisition, Conceptualization.

## Declaration of competing interest

The authors declare that they have no known competing financial interests or personal relationships that could have appeared to influence the work reported in this paper.

## Data Availability

No data was used for the research described in the article.

## Appendix A Supplementary data

Supplementary material 1 Image 1
