# Supplementary material for: The contribution of community transmission to the burden of hospital-associated pathogens: A systematic scoping review of epidemiological models
Source: One Health. 2024 Dec 16;20:100951. doi: 10.1016/j.onehlt.2024.100951 (PMC11733049; doi:10.1016/j.onehlt.2024.100951)
Supplement: Supplementary file 1 — Supplementary material 1 [file mmc1.docx]

Supplementary Materials:

The Contribution of Community Transmission to the Burden of Hospital-associated Pathogens: A Systematic Scoping Review of Epidemiological Models

Gary Lin, Suprena Poleon, Alisa Hamilton, Nalini Salvekar, Manuel Jara, Fardad Haghpanah, Cristina Lanzas, Ashley Hazel, Seth Blumberg, Suzanne Lenhart, Alun L. Lloyd, Anil Vullikanti, Eili Klein, For the CDC MInD Healthcare Network

Appendix A: Search Terms

# Search Strategy

The first phase was to collect all studies that included search terms based on three broad features: (1) Pathogens and Disease Type (2) Setting, and (3) methods. The definitions of the three features are listed below. We utilized Boolean search terms to query articles that contain at least one term from the three mentioned features. The initial search was conducted on three large databases: PubMed, Medline, Scopus, and Embase. All literature was collected and compiled using the Covidence Platform.

Once the databases of all the literature were collected, phase two (screening) was employed by reviewers from the CDC Modeling Infectious Disease (MInD) Network Community Transmission Working Group. The reviewers then read through all collected literature to determine whether pre-specific inclusion/exclusion criteria were met. **Figure S1** shows the proposed algorithm used to construct the search terms used to collect literature from the four databases.

**Figure S1.** The flow diagram illustrates the general flow in which we construct our search terms used to query the four databases.

# Search Terms and Definitions

1. **Infection Types:** Hospital acquired infections; HAI; Nosocomial infections; hospital infection; healthcare associated infections; HCAI; community-acquired infection; CAI
2. **Pathogen:** Extended-spectrum beta-lactamase; ESBL; Methicillin-resistant Staphylococcus aureus; MRSA; Clostridioides difficile; C. diff; Vancomycin-resistant Enterobacteriaceae; VRE; Candida auris; C. auris; Carbapenem-resistant Enterobacteriaceae; CRE; Carbapenem-resistant Acinetobacter; Microbial-drug-resistant; Antibiotic-resistant; Antimicrobial-resistant; Multidrug resistant
3. **Hospital (Acute Care):** Hospital; burn care hospital; community hospital; critical access hospital; field hospital; general hospital; geriatric hospital; high volume hospital; hospital ship; low volume hospital; magnet hospital; mental hospital; military hospital; non profit hospital; pediatric hospital; private hospital; public hospital; rural hospital; safety net hospital; teaching hospital; university hospital
   1. **Hospital components:** delivery room; dental clinic; heart center; hospital bed; hospital department; hospital food service; hospital laboratory; hospital pharmacy; kitchen; laundry; operating room; delivery room; dental clinic; burn unit; heart center; quick diagnosis unit; radiology department; recovery room; waiting room; ward; emergency ward; geriatric ward; high dependency unit; intensive care unit; intermediate care unit; maternity ward; nursing unit; observation unit; oncology ward; pediatric ward; psychiatric department; surgical ward
4. **Long Term Care Facilities:** Long term care; assisted living facility; hospice; nursing home rehabilitation center
5. **Ambulatory Care:** Ambulatory care; dental facility; cancer center; community mental health center; outpatient department; fertility clinic; health center; mental health center; pain clinic; poison center; sexually transmitted disease clinic; secondary care center; tertiary care center; student-run clinic; veterinary clinic; wellness center
6. **Medical Transport*:*** air medical transport; ambulance
7. **Banks:** Biobank; blood bank; bone bank; eye bank; milk bank; sperm bank; tissue bank
8. **Isolation facility**: isolation hospital; lazaret; leprosarium; quarantine station
9. **Laboratory**; clinical laboratory; core laboratory; dental laboratory; hospital laboratory
10. **Community surveillance:** Disease surveillance; epidemiological monitoring; wastewater-based epidemiology; epidemiological surveillance; public health surveillance; serological surveillance
11. **Residential:** residential home; household; dorms; community transmission
12. **Public Areas:** fitness center; morgue; pharmacy; restaurant; schools; universities
13. **Correctional Facilities:** Correctional facilities; halfway house; correctional facility; juvenile detention center; detention center
14. **Mechanistic/compartmental Modeling:** mathematical model; Compartmental model; disease simulation; theoretical model; computer simulation; discrete event simulation; population model; epidemiological model; ecological model; susceptible exposed infectious recovered model; susceptible infected recovered model; susceptible infected susceptible model; SEIR; SIR; Stochastic model; deterministic model; Dynamic transmission model; mechanistic models
15. **Agent based modeling:** agent based model; individual based; disease simulation; discrete event simulation
16. **Equation based modeling:** equation based; differential equation; ODE; stochastic ODE
17. **Spatial/network modeling:** cellular automaton; GIS; geographic information system; metapopulation; network model

# Search Query

Based on the definitions and word sets above, we conducted a search using the following combinations of word sets. The search queries that were executed on PubMed, Embase, and Scopus are outlined below.

- **[**(A) Infection Types **OR** (B) Pathogen**] AND [**(C) Hospital (Acute Care) **OR** (C)(1) Hospital Components **OR** (D) Long Term Care Facilities **OR** (E) Ambulatory Care **OR** (F) Medical Transport **OR** (G) Banks **OR** (H) Isolation Facility **OR** (I) Laboratory **OR** (J) Community Surveillance **OR** (K) Residential **OR** (L) Public Areas **OR** (M) Correctional Facilities**] AND [**(N) Mechanistic / compartmental modeling **OR** (O) Agent based modeling **OR** (P) Equation based modeling **OR** (Q) Spatial / network modeling**]**

**Example Embase Search Query:**

(('Hospital acquired infection*' OR 'HAI' OR 'Nosocomial infection*' OR 'hospital infection*' OR 'healthcare associated infection*' OR 'HCAI' OR 'community acquired infection' OR 'CAI')

OR

('Extended spectrum beta lactamase' OR 'ESBL' OR 'Methicillin resistant Staphylococcus aureus' OR 'MRSA' OR 'Clostridioides difficile' OR 'C. diff' OR 'Vancomycin resistant Enterobacteriaceae' OR 'VRE' OR 'Candida auris' OR 'C. auris' OR 'Carbapenem resistant Enterobacteriaceae' OR 'CRE' OR 'Carbapenem resistant Acinetobacter' OR 'Microbial drug resistant' OR 'Antibiotic resistant' OR 'Antimicrobial resistant' OR 'Multidrug resistant'))

AND

(('Acute care' OR 'hospital*' OR 'burn care hospital' OR 'community hospital*' OR 'critical access hospital*' OR 'field hospital*' OR 'general hospital*' OR 'geriatric hospital*' OR 'high volume hospital*' OR 'hospital ship*' OR 'low volume hospital*' OR 'magnet hospital*' OR 'mental hospital*' OR 'military hospital*' OR 'non profit hospital*' OR 'pediatric hospital*' OR 'private hospital*' OR 'public hospital*' OR 'rural hospital*' OR 'safety net hospital*' OR 'teaching hospita*' OR 'university hospital*')

OR

('delivery room*' OR 'dental clinic*' OR 'heart center*' OR 'hospital bed*' OR 'hospital department*' OR 'hospital food service*' OR 'hospital laborator*' OR 'hospital pharmac*' OR 'kitchen*' OR 'laundry*' OR 'operating room*' OR 'delivery room*' OR 'dental clinic*' OR 'burn unit*' OR 'heart center*' OR 'quick diagnosis unit*' OR 'radiology department*' OR 'recovery room*' OR 'waiting room*' OR 'ward*' OR 'emergency ward*' OR 'geriatric ward*' OR 'high dependency unit*' OR 'intensive care unit*' OR 'intermediate care unit*' OR 'maternity ward*' OR 'nursing unit*' OR 'observation unit*' OR 'oncology ward*' OR 'pediatric ward*' OR 'psychiatric department*' OR 'surgical ward*')

OR

('Long term care' OR 'assisted living facilit*' OR 'hospice' OR 'nursing home rehabilitation center*' OR 'convalescent')

OR

('Ambulatory care' OR 'dental facilit*' OR 'cancer center*' OR 'community mental health center*' OR 'outpatient department*' OR 'fertility clinic*' OR 'health center*' OR 'mental health center*' OR 'pain clinic*' OR 'poison center*' OR 'sexually transmitted disease clinic*' OR 'secondary care center*' OR 'tertiary care center*' OR 'student run clinic*' OR 'veterinary clinic*' OR 'wellness center*')

OR

('Disease surveillance' OR 'epidemiological monitoring' OR 'wastewater based epidemiology' OR 'epidemiological surveillance' OR 'public health surveillance' OR 'serological surveillance' OR 'communit*')

OR

('residential home*' OR 'household*' OR 'dorm*')

OR

('fitness center*' OR 'morgue*' OR 'pharmac*' OR 'restaurant*' OR 'school*' OR 'universit*')

OR

('Correctional facilit*' OR 'halfway house*' OR 'correctional facilit*' OR 'juvenile detention center*' OR 'detention center*'))

AND

(('mathematical model*' OR 'Compartmental model*' OR 'disease simulation*' OR 'theoretical model*' OR 'computer simulation*' OR 'computational model*' OR 'discrete event simulation*' OR 'population model*' OR 'epidemiological model*' OR 'ecological model*' OR 'susceptible exposed infectious recovered model*' OR 'susceptible infected recovered model*' OR 'susceptible infected susceptible model*' OR 'SEIR' OR 'SIR' OR 'Stochastic model*' OR 'deterministic model*' OR 'Dynamic transmission model*' OR 'mechanistic models' OR 'computational epidemiology')

OR

('agent based model*' OR 'individual based' OR 'disease simulation*' OR 'discrete event simulation*')

OR

('equation based' OR 'differential equation*' OR 'ODE' OR 'stochastic ODE')

OR

('cellular automaton' OR 'GIS' OR 'geographic information system' OR 'metapopulation' OR 'network model'))

Appendix B: Screening protocol

# Title and Abstract Screening: HAI Community Transmission Eligibility and Review Questions

*Inclusion Criteria*

1. Does the study relate to Hospital-acquired infections (HAI), healthcare-associated infections (HCAI), or community-acquired infections (CAI)? **If Yes, include; otherwise, exclude.**
2. Does the study include any of the mentioned pathogens: Extended-spectrum beta-lactamase (ESBL), Methicillin-resistant Staphylococcus aureus (MRSA), Clostridioides difficile (C. diff), Vancomycin-resistant Enterobacteriaceae (VRE), Candida Auris, Carbapenem-resistant Enterobacteriaceae (CRE), or Carbapenem-resistant Acinetobacter? **If Yes, include; otherwise, exclude.**
3. Does the study utilize mechanistic epidemiological models (e.g., mathematical model, computational model, network model, differential equations, agent-based model, discrete event simulation, compartmental model)? **If Yes, include; otherwise, exclude.**
4. Does the study include the community^[[1]](#footnote-2)^ in some capacity, which we will define as a setting outside of the acute care healthcare settings? **If Yes, include; otherwise, exclude.**
5. Does the study have interactions between hospitals/healthcare facilities and the community (e.g., movement flow)? **If Yes, include; otherwise, exclude.**

*Exclusion Criteria*

1. Is the study a systematic review? **If Yes, exclude; Otherwise, include.**
2. Is the study a conference paper or presentation? **If Yes, exclude; Otherwise, include.**
3. Does the model in the study include a fixed importation/exportation rate without community or LTCF settings depicted? **If Yes, exclude; Otherwise, include.**

Appendix C. Full Text Analysis Questions

*The goal is to figure out the current state of community transmission modeling.*

1. Which settings were modeled (select all that applies)?
   1. Acute Hospital
   2. Community – please specify (E.g., households, skilled nursing homes)
   3. Other -- Please specify
2. In which settings, mentioned above, was **transmission** modeled? Please specify.
3. Is “hospital and community interface” modeled? In other words, is the number of HAIs affected by the community prevalence of infection?
   1. Yes -- please specify which how hospital interact with the community.
   2. **No – exclude from study**
4. How many and which types of pathogens were included? Please specify the number of pathogens and type.
5. Were healthcare facilities included in the model (please select all that applies)?
   1. Long term care facilties -- please specify (E.g., skilled nursing homes, LTACHs, rehab/sub-acute)
   2. Ambulatory / Outpatient
   3. Acute-care hospitals / emergency rooms
   4. Other – please specify
6. What type of model was used in this study?
   1. Differential/Difference equation
   2. System Dynamics
   3. Agent-based model / Individual-based model
   4. Markov
   5. Hybrid – please specify simulation combination
   6. Other – please specify
7. Was the model stochastic and/or deterministic?
   1. Stochastic
   2. Deterministic
   3. Both
   4. Other – please specify
8. Was spatial information incorporated?
   1. Yes –please specify
   2. No
9. How many compartment states were in the model (susceptible, colonized, infected, recovered)?
   1. Yes –please specify
   2. No
10. Was travel behavior included? (e.g., movement between hospitals and communities)
    1. Please specify
11. What types of transmission included? (e.g., vector, zoonotic, indirect, foodborne, waterborne)
    1. Direct – please specify route (E.g. airborne/aersol, droplet)
    2. Environmental/fomite
    3. Vector/zoonotic
12. Are healthcare workers modeled explicitly?
    1. Yes – please specify type of HCW (e.g., nurses, primary physicians, physical therapists)
    2. No
13. Do healthcare workers have contact with patients in the model?
    1. Yes
    2. No
14. Are visitor interactions included in the healthcare setting?
    1. Yes
    2. No
15. How was data included in the model (Mark that all apply)?
    1. Parameterization
    2. Fitting to cases
    3. Initial Conditions
16. Can patients move between acute-care healthcare facilties and community?
    1. Yes
    2. No
17. Was the population heterogeneous?
    1. Yes – please specify whether the population was age structured, stratified by geographic or demographic, household type, etc.
    2. No
18. What was the flow between hospital and community? (e.g., one-way importation, bidirectional)
19. What aspect of the epidemiological dynamics were analyzed in the Results section? For example, did the analysis compare the prevalence during steady state/endemicity of different scenarios?
    1. Equilibrium/thresholds at which outbreaks dissipate or evolve
    2. Evolution
    3. Outbreak control
    4. Detection
    5. Steady-state/endemicity
    6. Other – please specify
20. What other aspects were considered in the study? Please specify the research outcome (e.g., cost, operation, targeted policy).
21. How were the parameters informed? (Mark all that apply)
    1. Data
    2. Expert judgment
    3. Monte carlo based methods
    4. Other – please specify
22. Is health equity/disparity studied in the model?
    1. Yes –please specify
    2. No
23. How was the model implemented (select all that applies)?
    1. Software – please specify (Anylogic, Arena, Vensim)
    2. Programming Languages – Please specify (Netlogo, Python, R)
    3. Other – please specify
24. Did the study include an economic analysis/cost-effectiveness study?
    1. Yes – please specify
    2. No
25. What kind of interventions were included? Please specify (e.g., Hand hygiene, Antibiotic stewardship, Isolation, HCW cohorting, Decolonization, Patient cohorting, Bundle, Active Surveillence, Screening strategies, Regional Registry).
26. What was the country of research? Please Specify (e.g., USA, not specified)

Appendix D: Full-text Analysis Results

**Table S1.** Title, Authors, Year, and Pathogens of Included Studies

| ***Study Number*** | ***Title*** | ***Authors*** | ***Year*** | ***Pathogens*** |
| --- | --- | --- | --- | --- |
| 1 | Quantifying Transmission of Clostridium difficile within and outside Healthcare Settings. | Durham et al. | 2016 | C.Diff |
| 2 | Modelling diverse sources of Clostridium difficile in the community: Importance of animals, infants and asymptomatic carriers | McLure et al. | 2019 | C.Diff |
| 3 | Seasonality and community interventions in a mathematical model of Clostridium difficile transmission | McLure et al. | 2019 | C.Diff |
| 4 | Creation of a Geospatially Explicit, Agent-based Model of a Regional Healthcare Network with Application to Clostridioides difficile Infection | Rhea et al. | 2019 | C.Diff |
| 5 | Modeling inpatient and outpatient antibiotic stewardship interventions to reduce the burden of Clostridioides difficile infection in a regional healthcare network | Rhea et al. | 2020 | C.Diff |
| 6 | Modeling the potential impact of administering vaccines against Clostridioides difficile infection to individuals in healthcare facilities | Toth et al. | 2020 | C.Diff |
| 7 | The projected effectiveness of Clostridium difficile vaccination as part of an integrated infection control strategy | van Kleef et al. | 2016 | C.Diff |
| 8 | Modelling of the transmission dynamics of carbapenem-resistant Klebsiella pneumoniae in hospitals and design of control strategies | Changruenngam et al. | 2022 | Carbapenem-resistant Klebsiella pneumonia |
| 9 | Knowing more of the iceberg: How detecting a greater proportion of carbapenem-resistant enterobacteriaceae carriers influences transmission | Bartsch et al. | 2020 | CRE |
| 10 | How introducing a registry with automated alerts for carbapenem-resistant enterobacteriaceae (CRE) may help control CRE spread in a region | Lee et al. | 2020 | CRE |
| 11 | How to Choose Target Facilities in a Region to Implement Carbapenem-resistant Enterobacteriaceae Control Measures | Lee et al. | 2021 | CRE |
| 12 | How Long-Term Acute Care Hospitals Can Play an Important Role in Controlling Carbapenem-Resistant Enterobacteriaceae in a Region: A Simulation Modeling Study | Lee et al. | 2021 | CRE |
| 13 | The Potential Trajectory of Carbapenem-Resistant Enterobacteriaceae, an Emerging Threat to Health-Care Facilities, and the Impact of the Centers for Disease Control and Prevention Toolkit. | Lee et al. | 2016 | CRE |
| 14 | Cost-effectiveness of carbapenem-resistant Enterobacteriaceae (CRE) surveillance in Maryland | Lin et al. | 2021 | CRE |
| 15 | The Potential for Interventions in a Long-term Acute Care Hospital to Reduce Transmission of Carbapenem-Resistant Enterobacteriaceae in Affiliated Healthcare Facilities | Toth et al. | 2017 | CRE |
| 16 | Quantifying where human acquisition of antibiotic resistance occurs: A mathematical modelling study | Knight et al. | 2018 | E.Coli |
| 17 | The relative impact of community and hospital antibiotic use on the selection of extended-spectrum beta-lactamase-producing Escherichia coli | MacFadden et al. | 2019 | E.Coli |
| 18 | Modelling the epidemiology of Escherichia coli ST131 and the impact of interventions on the community and healthcare centres | Talaminos et al. | 2016 | E.Coli |
| 19 | Does plasmid-based beta-lactam resistance increase E. coli infections: Modelling addition and replacement mechanisms | Godijk et al. | 2022 | ESBL-producing Enterobacteriaceae |
| 20 | Quantifying within-household transmission of extended-spectrum β-lactamase-producing bacteria | Haverkate et al. | 2017 | ESBL-producing Enterobacteriaceae |
| 21 | The impact of public health interventions on the future prevalence of ESBL-producing Klebsiella pneumoniae: a population based mathematical modelling study | Salazar-Vizcaya et al. | 2022 | ESBL‑producing Klebsiella pneumoniae |
| 22 | Modeling Interventions to Reduce the Spread of Multidrug-Resistant Organisms between Health Care Facilities in a Region | Bartsch et al. | 2021 | Generic nosocomial bacteria |
| 23 | Control of epidemics on hospital networks | Belik et al. | 2016 | Generic nosocomial bacteria |
| 24 | The Role of Nursing Homes in the Spread of Antimicrobial Resistance over the Healthcare Network | Van Den Dool et al. | 2016 | Generic nosocomial bacteria |
| 25 | Why sensitive bacteria are resistant to hospital infection control. | Van Kleef et al. | 2017 | Generic nosocomial bacteria |
| 26 | Quantifying the transmission dynamics of MRSA in the community and healthcare settings in a low-prevalence country: A modelling study | Di Ruscio et al. | 2019 | MRSA |
| 27 | Improving mathematical modeling of interventions to prevent healthcareassociated infections by interrupting transmission or pathogens: How common modeling assumptions about colonized individuals impact intervention effectiveness estimates | Gowler et al. | 2022 | MRSA |
| 28 | Dynamic contact networks of patients and MRSA spread in hospitals | Rocha et al. | 2020 | MRSA |
| 29 | Impact of inter-hospital transfers on the prevalence of resistant pathogens in a hospitalâ€“community system | Piotrowska et al. | 2020 | Generic MDRE |

**Table S3.** Model Characteristics and Structure of Included Studies

|  |  |  | **Model Characteristics and Structure** | | | | | | | |
| --- | --- | --- | --- | --- | --- | --- | --- | --- | --- | --- |
| ***Study Number*** | ***Authors*** | ***Year*** | ***Model*** | ***Stochastic or Deterministic*** | ***Programming Language*** | ***Number of compartment states*** | ***Differing Levels of Susceptibility*** | ***Differing Levels of Infectiousness*** | ***Multiple strains (Resistance and susceptible strains)*** | ***Differing levels of colonization detection*** |
| 1 | Durham et al. | 2016 | Stochastic Sim/Gillespie Algorithm | Stochastic | C++ | Equal to 3 | Yes | Yes | No | No |
| 2 | McLure et al. | 2019 | ABM/IBM | Stochastic | Not Specified | More than 3 | Yes | Yes | No | No |
| 3 | McLure et al. | 2019 | ABM/IBM | Stochastic | Not Specified | More than 3 | Yes | Yes | No | No |
| 4 | Rhea et al. | 2019 | ABM/IBM | Stochastic | Python | More than 3 | Yes | No | No | No |
| 5 | Rhea et al. | 2020 | ABM/IBM | Stochastic | Python | More than 3 | No | No | No | No |
| 6 | Toth et al. | 2020 | ABM/IBM | Stochastic | Not Specified | More than 3 | Yes | Yes | No | Yes |
| 7 | van Kleef et al. | 2016 | ABM/IBM | Stochastic | Not Specified | More than 3 | Yes | No | No | Yes |
| 8 | Changruenngam et al. | 2022 | Differential Equations | Deterministic | MATLAB | More than 3 | No | Yes | No | Yes |
| 9 | Bartsch et al. | 2020 | ABM/IBM | Stochastic | C++ | Less than 3 | No | No | No | Yes |
| 10 | Lee et al. | 2020 | ABM/IBM | Stochastic | C++ | Less than 3 | No | Yes | No | Yes |
| 11 | Lee et al. | 2021 | ABM/IBM | Stochastic | C++ | Less than 3 | No | No | No | Yes |
| 12 | Lee et al. | 2021 | ABM/IBM | Stochastic | C++ | Less than 3 | No | No | No | Yes |
| 13 | Lee et al. | 2016 | ABM/IBM | Stochastic | C++ | Less than 3 | No | No | No | Yes |
| 14 | Lin et al. | 2021 | Differential Equations | Deterministic | Python | More than 3 | Yes | No | No | Yes |
| 15 | Toth et al. | 2017 | ABM/IBM | Stochastic | Not Specified | Less than 3 | Yes | No | No | Yes |
| 16 | Knight et al. | 2018 | Differential Equations | Stochastic | R | Equal to 3 | No | Yes | Yes | No |
| 17 | MacFadden et al. | 2019 | Differential Equations | Deterministic | R | More than 3 | Yes | No | Yes | No |
| 18 | Talaminos et al. | 2016 | Discrete Event/Microsim | Stochastic | Not Specified | More than 3 | Yes | No | No | No |
| 19 | Godijk et al. | 2022 | Differential Equations | Deterministic | Not Specified | More than 3 | Yes | Yes | Yes | No |
| 20 | Haverkate et al. | 2017 | Markov Model | Deterministic | Mathematica | Less than 3 | Yes | Yes | No | No |
| 21 | Salazar-Vizcaya et al. | 2022 | Differential Equations | Deterministic | R | More than 3 | Yes | Yes | No | No |
| 22 | Bartsch et al. | 2021 | ABM/IBM | Stochastic | C++ | Less than 3 | No | No | No | No |
| 23 | Belik et al. | 2016 | ABM/IBM | Stochastic | Not Specified | Less than 3 | No | No | No | No |
| 24 | Van Den Dool et al. | 2016 | Discrete Event/Microsim | Stochastic | Not Specified | Less than 3 | No | No | No | No |
| 25 | Van Kleef et al. | 2017 | Differential Equations | Deterministic | R | Equal to 3 | No | No | Yes | No |
| 26 | Di Ruscio et al. | 2019 | ABM/IBM | Stochastic | Not Specified | Equal to 3 | No | No | No | No |
| 27 | Gowler et al. | 2022 | Differential Equations | Deterministic | R | Equal to 3 | No | Yes | No | Yes |
| 28 | Rocha et al. | 2020 | Network Sim | Stochastic | Not Specified | Equal to 3 | No | No | No | Yes |
| 29 | Piotrowska et al. | 2020 | Network Sim | Deterministic | Python | Less than 3 | No | No | No | Yes |

**Table S3.** Model Settings and Country of Included Studies.

|  |  |  | **Model Setting** | | | |
| --- | --- | --- | --- | --- | --- | --- |
| ***Study Number*** | ***Authors*** | ***Year*** | ***Short-term Acute Care Hospital*** | ***Long Term Healthcare Facilities*** | ***Other Community not including LTCF and NH*** | ***Country*** |
| 1 | Durham et al. | 2016 | Yes | Yes | Yes | USA |
| 2 | McLure et al. | 2019 | Yes | No | Yes | USA |
| 3 | McLure et al. | 2019 | Yes | No | Yes | USA |
| 4 | Rhea et al. | 2019 | Yes | Yes | Yes | USA |
| 5 | Rhea et al. | 2020 | Yes | Yes | No | USA |
| 6 | Toth et al. | 2020 | Yes | Yes | Yes | USA |
| 7 | van Kleef et al. | 2016 | Yes | Yes | Yes | UK |
| 8 | Changruenngam et al. | 2022 | Yes | No | Yes | Not specified |
| 9 | Bartsch et al. | 2020 | Yes | Yes | No | USA |
| 10 | Lee et al. | 2020 | Yes | Yes | Yes | USA |
| 11 | Lee et al. | 2021 | Yes | Yes | No | USA |
| 12 | Lee et al. | 2021 | Yes | Yes | No | USA |
| 13 | Lee et al. | 2016 | Yes | Yes | No | USA |
| 14 | Lin et al. | 2021 | Yes | Yes | Yes | USA |
| 15 | Toth et al. | 2017 | Yes | Yes | No | USA |
| 16 | Knight et al. | 2018 | Yes | No | Yes | UK |
| 17 | MacFadden et al. | 2019 | Yes | No | Yes | Sweden |
| 18 | Talaminos et al. | 2016 | Yes | Yes | Yes | Spain |
| 19 | Godijk et al. | 2022 | Yes | No | Yes | Netherlands |
| 20 | Haverkate et al. | 2017 | Yes | No | Yes | Netherlands |
| 21 | Salazar-Vizcaya et al. | 2022 | Yes | No | Yes | Switzerland |
| 22 | Bartsch et al. | 2021 | Yes | Yes | No | USA |
| 23 | Belik et al. | 2016 | Yes | No | Yes | Germany |
| 24 | Van Den Dool et al. | 2016 | Yes | Yes | Yes | Netherlands |
| 25 | Van Kleef et al. | 2017 | Yes | No | Yes | EU |
| 26 | Di Ruscio et al. | 2019 | Yes | Yes | Yes | Norway |
| 27 | Gowler et al. | 2022 | Yes | No | Yes | Not specified |
| 28 | Rocha et al. | 2020 | Yes | Yes | No | Sweden |
| 29 | Piotrowska et al. | 2020 | Yes | No | Yes | Germany |

**Table S4.** Parameterization Methods of Included Studies

|  |  |  | **Parameterization** | | | | |
| --- | --- | --- | --- | --- | --- | --- | --- |
| ***Study Number*** | ***Authors*** | ***Year*** | ***Parameters calibrated or fitted to data*** | ***Data Informed*** | ***Sensitivity analysis conducted*** | ***Parameters based on other literature*** | ***Expert judgement*** |
| 1 | Durham et al. | 2016 | Yes | Yes | Yes | Yes | Yes |
| 2 | McLure et al. | 2019 | Yes | Yes | Yes | Yes | Yes |
| 3 | McLure et al. | 2019 | Yes | Yes | Yes | Yes | No |
| 4 | Rhea et al. | 2019 | Yes | Yes | No | Yes | Yes |
| 5 | Rhea et al. | 2020 | Yes | Yes | No | Yes | Yes |
| 6 | Toth et al. | 2020 | Yes | Yes | Yes | Yes | Yes |
| 7 | van Kleef et al. | 2016 | Yes | Yes | Yes | Yes | Yes |
| 8 | Changruenngam et al. | 2022 | Yes | No | No | Yes | Yes |
| 9 | Bartsch et al. | 2020 | No | Yes | Yes | Yes | Yes |
| 10 | Lee et al. | 2020 | No | Yes | Yes | Yes | Yes |
| 11 | Lee et al. | 2021 | No | Yes | Yes | Yes | Yes |
| 12 | Lee et al. | 2021 | No | Yes | Yes | Yes | Yes |
| 13 | Lee et al. | 2016 | No | Yes | Yes | Yes | Yes |
| 14 | Lin et al. | 2021 | No | Yes | Yes | Yes | Yes |
| 15 | Toth et al. | 2017 | Yes | Yes | Yes | Yes | Yes |
| 16 | Knight et al. | 2018 | Yes | Yes | Yes | Yes | Yes |
| 17 | MacFadden et al. | 2019 | Yes | Yes | Yes | Yes | Yes |
| 18 | Talaminos et al. | 2016 | Yes | No | Yes | Yes | Yes |
| 19 | Godijk et al. | 2022 | No | Yes | No | Yes | No |
| 20 | Haverkate et al. | 2017 | Yes | Yes | Yes | Yes | Yes |
| 21 | Salazar-Vizcaya et al. | 2022 | Yes | Yes | Yes | Yes | Yes |
| 22 | Bartsch et al. | 2021 | No | Yes | Yes | Yes | Yes |
| 23 | Belik et al. | 2016 | No | Yes | No | No | No |
| 24 | Van Den Dool et al. | 2016 | No | Yes | Yes | Yes | Yes |
| 25 | Van Kleef et al. | 2017 | No | Yes | Yes | Yes | Yes |
| 26 | Di Ruscio et al. | 2019 | Yes | Yes | Yes | Yes | No |
| 27 | Gowler et al. | 2022 | Yes | Yes | Yes | Yes | Yes |
| 28 | Rocha et al. | 2020 | No | Yes | No | Yes | No |
| 29 | Piotrowska et al. | 2020 | No | Yes | No | Yes | No |

**Table S5.** Modeled Interventions of Included Studies

|  |  |  | **Modeled Interventions** | | | | | | | | | | |
| --- | --- | --- | --- | --- | --- | --- | --- | --- | --- | --- | --- | --- | --- |
| ***Study Number*** | ***Authors*** | ***Year*** | ***Varying antimicrobial consumption and prescribing*** | ***Non-specified hospital transmission reduction*** | ***Non-specified community transmission reduction*** | ***Improve screening and surveillance*** | ***Improve HCW hygiene*** | ***Implement contact precautions and isolation*** | ***Environmental cleaning*** | ***Interfacility coordination*** | ***Decolonization treatment*** | ***Regional registry*** | ***Vaccine*** |
| 1 | Durham et al. | 2016 | No | No | Yes | Yes | Yes | Yes | Yes | No | No | No | No |
| 2 | McLure et al. | 2019 | No | No | No | No | No | No | No | No | No | No | No |
| 3 | McLure et al. | 2019 | Yes | Yes | Yes | No | No | No | No | No | No | No | No |
| 4 | Rhea et al. | 2019 | No | No | No | No | No | No | No | No | No | No | No |
| 5 | Rhea et al. | 2020 | Yes | No | No | No | No | No | No | No | No | No | No |
| 6 | Toth et al. | 2020 | No | No | No | No | No | No | No | No | No | No | Yes |
| 7 | van Kleef et al. | 2016 | Yes | No | No | No | No | No | No | No | No | No | Yes |
| 8 | Changruenngam et al. | 2022 | Yes | No | No | No | No | Yes | No | No | No | No | No |
| 9 | Bartsch et al. | 2020 | No | No | No | Yes | No | Yes | No | Yes | No | No | No |
| 10 | Lee et al. | 2020 | No | No | No | No | No | No | No | No | No | Yes | No |
| 11 | Lee et al. | 2021 | No | Yes | No | No | No | No | No | No | No | Yes | No |
| 12 | Lee et al. | 2021 | No | No | No | Yes | Yes | Yes | No | No | Yes | No | No |
| 13 | Lee et al. | 2016 | No | No | No | Yes | No | Yes | No | Yes | No | No | No |
| 14 | Lin et al. | 2021 | No | Yes | No | Yes | No | No | No | No | No | No | No |
| 15 | Toth et al. | 2017 | No | No | No | Yes | No | Yes | No | No | No | No | No |
| 16 | Knight et al. | 2018 | No | No | No | No | No | No | No | No | No | No | No |
| 17 | MacFadden et al. | 2019 | Yes | No | No | No | No | No | No | No | No | No | No |
| 18 | Talaminos et al. | 2016 | Yes | Yes | No | No | No | No | No | No | No | No | No |
| 19 | Godijk et al. | 2022 | Yes | No | No | No | No | No | No | No | No | No | No |
| 20 | Haverkate et al. | 2017 | No | No | No | No | No | No | No | No | No | No | No |
| 21 | Salazar-Vizcaya et al. | 2022 | Yes | Yes | No | No | No | No | No | No | No | No | No |
| 22 | Bartsch et al. | 2021 | No | No | No | No | No | Yes | No | Yes | Yes | No | No |
| 23 | Belik et al. | 2016 | No | No | No | Yes | No | No | No | No | No | No | No |
| 24 | Van Den Dool et al. | 2016 | No | No | No | No | No | No | No | No | No | No | No |
| 25 | Van Kleef et al. | 2017 | No | No | No | No | Yes | No | No | No | No | No | No |
| 26 | Di Ruscio et al. | 2019 | No | No | No | Yes | No | Yes | No | No | Yes | No | No |
| ***Study Number*** | ***Authors*** | ***Year*** | ***Varying antimicrobial consumption and prescribing*** | ***Non-specified hospital transmission reduction*** | ***Non-specified community transmission reduction*** | ***Improve screening and surveillance*** | ***Improve HCW hygiene*** | ***Implement contact precautions and isolation*** | ***Environmental cleaning*** | ***Interfacility coordination*** | ***Decolonization treatment*** | ***Regional registry*** | ***Vaccine*** |
| 27 | Gowler et al. | 2022 | No | Yes | No | No | Yes | No | No | No | Yes | No | No |
| 28 | Rocha et al. | 2020 | No | No | No | Yes | Yes | No | No | No | No | No | No |
| 29 | Piotrowska et al. | 2020 | No | No | No | No | No | No | No | No | No | No | No |

**Table S6.** Model Population Characteristics of Included Studies

|  |  |  | **Population Characteristics** | | |
| --- | --- | --- | --- | --- | --- |
| ***Study Number*** | ***Authors*** | ***Year*** | ***Healthcare Workers*** | ***Visitors*** | ***Population stratification*** |
| 1 | Durham et al. | 2016 | None | None | Age |
| 2 | McLure et al. | 2019 | None | None | None |
| 3 | McLure et al. | 2019 | None | None | None |
| 4 | Rhea et al. | 2019 | None | None | Gender, age, race, and country of residence |
| 5 | Rhea et al. | 2020 | None | None | Gender, age, race, and country of residence |
| 6 | Toth et al. | 2020 | None | None | None |
| 7 | van Kleef et al. | 2016 | None | None | None |
| 8 | Changruenngam et al. | 2022 | HCW are vectors | None | None |
| 9 | Bartsch et al. | 2020 | None | None | None |
| 10 | Lee et al. | 2020 | None | None | None |
| 11 | Lee et al. | 2021 | None | None | None |
| 12 | Lee et al. | 2021 | None | None | None |
| 13 | Lee et al. | 2016 | None | None | None |
| 14 | Lin et al. | 2021 | None | None | None |
| 15 | Toth et al. | 2017 | None | None | None |
| 16 | Knight et al. | 2018 | None | None | None |
| 17 | MacFadden et al. | 2019 | None | None | None |
| 18 | Talaminos et al. | 2016 | None | None | None |
| 19 | Godijk et al. | 2022 | None | None | None |
| 20 | Haverkate et al. | 2017 | None | Yes | Age, Gender |
| 21 | Salazar-Vizcaya et al. | 2022 | None | None | None |
| 22 | Bartsch et al. | 2021 | None | None | None |
| 23 | Belik et al. | 2016 | None | None | None |
| 24 | Van Den Dool et al. | 2016 | None | None | None |
| 25 | Van Kleef et al. | 2017 | HCW mix with patients | None | None |
| 26 | Di Ruscio et al. | 2019 | HCW mix with patients | None | Age, Ethnicity, Occupation |
| 27 | Gowler et al. | 2022 | None | None | Age |
| 28 | Rocha et al. | 2020 | None | None | None |
| 29 | Piotrowska et al. | 2020 | None | None | state of residence |

**Table S7.** Modeling Transmission and Movement Assumptions of Included Studies

|  |  |  | **Transmission and Movement** | | | |
| --- | --- | --- | --- | --- | --- | --- |
| ***Study Number*** | ***Authors*** | ***Year*** | ***Transmission Type*** | ***Hospital-community interactions*** | ***Spatial Features*** | ***Travel Behavior*** |
| 1 | Durham et al. | 2016 | Direct | Admission and discharge | None | No |
| 2 | McLure et al. | 2019 | Direct, Zoonotic, and Foodborne | Admission and discharge | None | None |
| 3 | McLure et al. | 2019 | Direct | Admission and discharge | None | No |
| 4 | Rhea et al. | 2019 | Not specified | Admission, discharge, transfer, and readmission | Spatially Defined Locations | Movement between hospitals and community |
| 5 | Rhea et al. | 2020 | Not specified | Admission, discharge, transfer, and readmission | Spatially Defined Locations | Movement between hospitals and community |
| 6 | Toth et al. | 2020 | Direct | Admission, discharge, transfer, and readmission | None | Between hospitals |
| 7 | van Kleef et al. | 2016 | Direct | Admission, discharge, and readmission | None | No |
| 8 | Changruenngam et al. | 2022 | Direct | Admission and discharge | None | None |
| 9 | Bartsch et al. | 2020 | Fomite and HCW-mediated | Admission, discharge, transfer, and readmission | None | Between hospitals |
| 10 | Lee et al. | 2020 | Direct | Admission, discharge, transfer, and readmission | None | None |
| 11 | Lee et al. | 2021 | Direct | Admission, discharge, transfer, and readmission | None | Between hospitals |
| 12 | Lee et al. | 2021 | Direct | Admission, discharge, transfer, and readmission | None | Between hospitals |
| 13 | Lee et al. | 2016 | Direct | Admission, discharge, transfer, and readmission | None | No |
| 14 | Lin et al. | 2021 | Direct | Admission, discharge, and transfer | Spatially Defined Locations | Movement between hospitals and community |
| 15 | Toth et al. | 2017 | Direct | Admission, discharge, transfer, and readmission | None | No |
| 16 | Knight et al. | 2018 | Direct | Admission and discharge | None | No |
| 17 | MacFadden et al. | 2019 | Direct | Admission and discharge | None | No |
| 18 | Talaminos et al. | 2016 | Direct | Admission and discharge | None | None |
| 19 | Godijk et al. | 2022 | Not specified | Admission, discharge, and readmission | None | None |
| 20 | Haverkate et al. | 2017 | Direct | Admission and discharge | None | Travel between high and low prevlance regions |
| ***Study Number*** | ***Authors*** | ***Year*** | ***Transmission Type*** | ***Hospital-community interactions*** | ***Spatial Features*** | ***Travel Behavior*** |
| 21 | Salazar-Vizcaya et al. | 2022 | Direct | Admission and discharge | None | Travel between high and low prevlance regions |
| 22 | Bartsch et al. | 2021 | Fomite and HCW-mediated | Admission, discharge, transfer, and readmission | None | Between hospitals |
| 23 | Belik et al. | 2016 | Direct | Admission and Discharge | None | None |
| 24 | Van Den Dool et al. | 2016 | Direct | Admission, discharge, and transfer | Proximity based movement | No |
| 25 | Van Kleef et al. | 2017 | HCW-mediated | Admission and discharge | None | No |
| 26 | Di Ruscio et al. | 2019 | Direct | Admission and discharge | Proximity based movement | International and local travel was simulated |
| 27 | Gowler et al. | 2022 | Not specified | Admission and discharge | None | None |
| 28 | Rocha et al. | 2020 | Direct | Admission and Discharge | Spatially Defined Locations | Between hospitals |
| 29 | Piotrowska et al. | 2020 | Direct | Admission, discharge, and readmission | Spatially Defined Locations | None |

**Table S8.** Role of Data in Model of Included Studies

|  |  |  | **Role of Data** | | | |
| --- | --- | --- | --- | --- | --- | --- |
| ***Study Number*** | ***Authors*** | ***Year*** | ***Contact or Movement Network*** | ***Informing Parameters*** | ***Calibration/Fitting*** | ***Quantifying Uncertainty*** |
| 1 | Durham et al. | 2016 | No | Yes | Yes | Yes |
| 2 | McLure et al. | 2019 | No | Yes | Yes | No |
| 3 | McLure et al. | 2019 | No | Yes | Yes | No |
| 4 | Rhea et al. | 2019 | Yes | Yes | Yes | No |
| 5 | Rhea et al. | 2020 | Yes | Yes | Yes | No |
| 6 | Toth et al. | 2020 | No | Yes | Yes | No |
| 7 | van Kleef et al. | 2016 | No | Yes | Yes | No |
| 8 | Changruenngam et al. | 2022 | No | Yes | Yes | No |
| 9 | Bartsch et al. | 2020 | Yes | Yes | Yes | No |
| 10 | Lee et al. | 2020 | No | Yes | Yes | No |
| 11 | Lee et al. | 2021 | Yes | Yes | Yes | No |
| 12 | Lee et al. | 2021 | Yes | Yes | Yes | No |
| 13 | Lee et al. | 2016 | Yes | Yes | Yes | No |
| 14 | Lin et al. | 2021 | Yes | Yes | No | No |
| 15 | Toth et al. | 2017 | Yes | Yes | Yes | No |
| 16 | Knight et al. | 2018 | No | Yes | Yes | No |
| 17 | MacFadden et al. | 2019 | No | Yes | Yes | No |
| 18 | Talaminos et al. | 2016 | No | Yes | Yes | No |
| 19 | Godijk et al. | 2022 | No | Yes | No | No |
| 20 | Haverkate et al. | 2017 | No | Yes | Yes | No |
| 21 | Salazar-Vizcaya et al. | 2022 | No | Yes | Yes | No |
| 22 | Bartsch et al. | 2021 | Yes | Yes | Yes | No |
| 23 | Belik et al. | 2016 | Yes | Yes | No | No |
| 24 | Van Den Dool et al. | 2016 | Yes | Yes | No | No |
| 25 | Van Kleef et al. | 2017 | No | Yes | No | No |
| 26 | Di Ruscio et al. | 2019 | Yes | Yes | Yes | No |
| 27 | Gowler et al. | 2022 | No | Yes | Yes | Yes |
| 28 | Rocha et al. | 2020 | Yes | Yes | No | No |
| 29 | Piotrowska et al. | 2020 | Yes | Yes | No | No |

**Table S9.** Study Measures and Outcomes of Included Studies

|  |  |  | **Study Measures and Outcomes** | | | |
| --- | --- | --- | --- | --- | --- | --- |
| ***Study Number*** | ***Authors*** | ***Year*** | ***Observed Epidemiological Dynamics*** | ***Study Outcomes*** | ***Health Equity Analysis*** | ***Cost-effectiveness study*** |
| 1 | Durham et al. | 2016 | Total transmissions | Incidence rate | No | No |
| 2 | McLure et al. | 2019 | Equilibirum/steady-state | Reproductive Number | No | No |
| 3 | McLure et al. | 2019 | Evolution/trajecotry | Incidence, Prevalence, and Transmission rate | No | No |
| 4 | Rhea et al. | 2019 | Total counts | incidence of C difficile infection | No | No |
| 5 | Rhea et al. | 2020 | Total counts | Incidence | No | No |
| 6 | Toth et al. | 2020 | Evolution/trajecotry | Incidence | No | No |
| 7 | van Kleef et al. | 2016 | Total counts | Averted cases | No | No |
| 8 | Changruenngam et al. | 2022 | Equilibrium/steady-state, Evolution/trajectory | Prevalencce and reproductive number | No | No |
| 9 | Bartsch et al. | 2020 | Evolution/trajecotry | Prevalence | No | No |
| 10 | Lee et al. | 2020 | Incidence and Prevalence | Averted cases | No | No |
| 11 | Lee et al. | 2021 | Evolution/trajecotry | Prevalence | No | No |
| 12 | Lee et al. | 2021 | Incidence and Prevalence | Averted cases and deaths and cost effectiveness | No | No |
| 13 | Lee et al. | 2016 | Evolution/trajecotry | CRE prevlance over time | No | No |
| 14 | Lin et al. | 2021 | Incidence or total count? | Averted cases and cost effectiveness | No | Yes |
| 15 | Toth et al. | 2017 | Equilibrium/steady-state and total average | Range of average colonization prevalence and total transmission, Reproductive Number | No | No |
| 16 | Knight et al. | 2018 | Equilibirum/steady-state | Proportion of total resistance in the hospital | No | No |
| 17 | MacFadden et al. | 2019 | Equilibrium/steady-state, Evolution/trajectory | Colonization prevalence in community and hospitals | No | No |
| 18 | Talaminos et al. | 2016 | Total transmissions | Incidence over two years | No | No |
| 19 | Godijk et al. | 2022 | Incidence; Equilibrium? | Survival of resistant or non-resistant strain | No | No |
| ***Study Number*** | ***Authors*** | ***Year*** | ***Observed Epidemiological Dynamics*** | ***Study Outcomes*** | ***Health Equity Analysis*** | ***Cost-effectiveness study*** |
| 20 | Haverkate et al. | 2017 | Equilibirum/steady-state | Prevalence rate | No | No |
| 21 | Salazar-Vizcaya et al. | 2022 | Evolution/trajecotry | Colonization prevalence in community and hospitals | No | No |
| 22 | Bartsch et al. | 2021 | Evolution/trajecotry | Prevalence and incidence | No | No |
| 23 | Belik et al. | 2016 | Equilibrium/steady-state | Equilibirum, effect of screening to reduce endemic prevalence level | No | No |
| 24 | Van Den Dool et al. | 2016 | Equilibrium/steady-state, Evolution/trajectory | Number colonized | No | No |
| 25 | Van Kleef et al. | 2017 | Equilibirum/steady-state | Annual incidence rate ratios (IRR) | No | No |
| 26 | Di Ruscio et al. | 2019 | Total transmissions | Average number of transmission events by age of the infector andof the infected (transmission matrix). infections by setting of acquisition | No | No |
| 27 | Gowler et al. | 2022 | transmission rate, decolonization rate, and both simultaneously | Averted cases | No | No |
| 28 | Rocha et al. | 2020 | Incidence or total count? | Incidence and Transmission rate | No | No |
| 29 | Piotrowska et al. | 2020 | Equilibrium/steady-state, Evolution/trajectory | Prevalence | No | No |

**Table S10.** Model summary of Included Studies.

| ***Study Number*** | ***Authors*** | ***Year*** | ***Summary*** |
| --- | --- | --- | --- |
| 1 | Durham et al. | 2016 | The model is a mechanistic framework that simulates Clostridium difficile transmission within hospitals, long-term care facilities, and the community, accounting for interactions between these settings. It incorporates transitions based on colonization, infection status, and movement between environments, emphasizing the role of asymptomatic carriers and community sources in hospital-onset CDI. Using national data for calibration, it evaluates the impact of interventions like reducing transmission or antibiotic use on CDI incidence and prevalence. |
| 2 | McLure et al. | 2019 | The mathematical model assesses Clostridium difficile transmission dynamics across hospitals, communities, infants, adults, and animal reservoirs, focusing on how each contributes to the pathogen’s persistence. It incorporates compartmental structures for colonized, infected, and susceptible individuals and calculates reproduction numbers for different settings. The findings show hospital transmission alone cannot sustain the pathogen (reproduction number <1), while community transmission is sufficient (reproduction number >1) in most scenarios, even without animal reservoirs. Infants contribute significantly to community transmission (17%), and even small contributions from animal reservoirs (>3.5–26.0% of exposures) could sustain transmission. This highlights the importance of addressing community sources, asymptomatic carriers, and potential animal reservoirs in control strategies. |
| 3 | McLure et al. | 2019 | The mathematical model described in the article evaluates Clostridium difficile (CDI) transmission dynamics within hospitals and surrounding communities, accounting for seasonality and the impact of various interventions. It features a compartmental structure that differentiates individuals by settings (hospital or community), colonization and infection status, gut flora condition, immune response, and age (with infants modeled separately). The model also includes seasonal variations in antibiotic prescriptions and transmission rates to replicate observed seasonal CDI patterns. |
| 4 | Rhea et al. | 2019 | The study details the development of a geospatially explicit, agent-based model (ABM) to simulate the movement and disease dynamics of a synthetic population within a regional healthcare network in North Carolina, with an application to Clostridioides difficile infection (CDI). The model includes agents representing the population, incorporating demographic, spatial, and healthcare interaction data, allowing simulation of patient movement across hospitals, nursing homes, long-term acute care hospitals, and community settings. It employs a disease-specific submodel to capture CDI natural history, including colonization, infection, and recurrence dynamics, considering risk factors like antibiotic exposure, age, and healthcare settings. |
| 5 | Rhea et al. | 2020 | The article investigates the impact of antibiotic stewardship interventions on reducing Clostridioides difficile infection (CDI) within a regional healthcare network in North Carolina using a geospatially explicit agent-based model. It examines scenarios targeting reductions in unnecessary antibiotic prescribing and inappropriate selection across inpatient (hospitals, nursing homes) and outpatient settings. A modeled 30% reduction in antibiotics resulted in significant decreases in CDI incidence, including a 17% reduction in healthcare-onset CDI and a 7% reduction in community-associated CDI, highlighting the effectiveness of broad, network-wide interventions. |
| 6 | Toth et al. | 2020 | The agent-based model simulates Clostridioides difficile transmission within a regional healthcare network, capturing patient movement among hospitals, nursing homes, and the community. It models transitions between disease states, such as susceptibility, colonization, and symptomatic CDI, with calibrated parameters reflecting real-world infection and transmission dynamics. Symptomatic CDI cases are modeled as 30 times more transmissible than asymptomatic carriers, with contact precautions reducing transmission by half. The model incorporates a simulated vaccination campaign targeting progression to symptomatic CDI, exploring varying vaccine efficacies, immunity durations, and vaccination rates among discharged patients. Results highlight significant potential for reducing CDI cases, particularly in high-transmission healthcare settings, through both direct protection and indirect benefits. |
| 7 | van Kleef et al. | 2016 | The model is a dynamic transmission framework that simulates Clostridium difficile spread in an ICU setting, incorporating patient movement between hospitals, communities, and long-term care facilities (LTCFs). It tracks transitions between protected, susceptible, colonized, and infected states, with transmission driven by interactions with symptomatic and asymptomatic carriers, contaminated environments, and staff. Vaccination is modeled to prevent progression to symptomatic CDI but not colonization, with strategies targeting CDI-history patients, LTCF residents, elective surgery patients, or all groups combined. Sensitivity analyses explore varying vaccine efficacy, asymptomatic transmission rates, and antimicrobial use, revealing the interaction of these factors in driving CDI dynamics. The model emphasizes vaccination's role as a complementary strategy to infection control, particularly in high-transmission or high-antimicrobial-use settings. |
| 8 | Changruenngam et al. | 2022 | The model examines the transmission dynamics of carbapenem-resistant Klebsiella pneumoniae (CRKP) within healthcare units, focusing on patient and staff interactions that drive the spread. Patients are classified into states like uncolonized, asymptomatic colonized, symptomatic colonized, and relapsed, with subcategories for multiple treatment failures or relapses. Transmission occurs mainly through contaminated staff, with factors like contact precautions and decontamination affecting rates. The model highlights the role of antibiotic treatment efficacy, combining treatment success probabilities and durations, in shaping infection prevalence. Admission of colonized patients significantly contributes to CRKP persistence, and the basic reproduction number (R₀) provides insights into controllability under various scenarios. Simulations reveal that effective treatment and strict precaution compliance are critical for reducing transmission, with intervention strategies tailored to specific drivers like symptomatic cases or asymptomatic carriers. This comprehensive approach aims to guide targeted infection control policies in hospitals. |
| 9 | Bartsch et al. | 2020 | The model is an agent-based simulation framework representing the healthcare ecosystem in Orange County, California, designed to assess the impact of detecting and isolating carbapenem-resistant Enterobacteriaceae (CRE) carriers. It incorporates 102 healthcare facilities, including acute care hospitals, long-term acute care hospitals (LTACHs), and nursing homes, simulating patient movements and CRE transmission over a 10-year period. Patients are represented as agents with individual decision-making processes, interacting dynamically with facilities and other agents. The model evaluates scenarios of increasing CRE detection levels, varying contact precaution effectiveness, and interfacility communication rates, to determine thresholds for reducing CRE prevalence. It emphasizes the critical role of LTACHs in driving countywide transmission, highlighting the need for targeted interventions in high-risk facilities. |
| 10 | Lee et al. | 2020 | The model is an agent-based simulation of the healthcare ecosystem in the Chicago metropolitan area, representing patient movements and interactions across 462 facilities, including hospitals, long-term acute care hospitals, and nursing facilities. It evaluates the impact of implementing an electronic registry to track carbapenem-resistant Enterobacteriaceae (CRE) carriers, enabling targeted infection prevention measures. Patients are modeled as agents with individual decisions about movements, admissions, and discharge patterns, interacting within and between facilities. The model tests various registry participation levels and explores the resulting reductions in CRE transmission and prevalence across the region. |
| 11 | Lee et al. | 2021 | The model is an agent-based simulation of the healthcare ecosystem in the Chicago metropolitan area, representing patient movement and interactions across 462 healthcare facilities, including hospitals, long-term acute care hospitals, and skilled nursing facilities. It evaluates the impact of various facility-targeting strategies for implementing CRE (carbapenem-resistant Enterobacteriaceae) prevention bundles and an electronic registry. Patients are modeled as agents with individualized behaviors, allowing the simulation of transmission dynamics and the impact of interventions across interconnected facilities. The model explores the effectiveness of targeting facilities based on metrics such as bed size, patient transfer patterns, length of stay, or combined metrics, to optimize reductions in CRE transmission and prevalence while considering economic and practical constraints. |
| 12 | Lee et al. | 2021 | The model is an agent-based simulation of the healthcare ecosystem in the Chicago metropolitan area, representing the interconnected movement of patients across 462 facilities, including long-term acute care hospitals (LTACHs), acute care hospitals, and nursing facilities. It assesses the impact of implementing a prevention bundle targeting carbapenem-resistant Enterobacteriaceae (CRE) in LTACHs and/or intensive care units (ICUs). Patients are modeled as agents who can carry or not carry CRE, with transmission occurring within facilities and across the healthcare network. The model evaluates the effectiveness of targeted interventions in reducing CRE prevalence, transmission, and associated healthcare costs across the region. It highlights the pivotal role of LTACHs in CRE control, showing substantial regional benefits even with interventions in a small number of high-prevalence facilities. |
| 13 | Lee et al. | 2016 | The model uses the Regional Healthcare Ecosystem Analyst (RHEA) framework to simulate the spread and control of carbapenem-resistant Enterobacteriaceae (CRE) in Orange County, California. It includes detailed representations of acute-care hospitals, long-term acute-care hospitals (LTACs), and nursing homes, modeling patient movements and CRE transmission across these facilities and the community. The model evaluates the impact of different control strategies, including facility-level and coordinated regional interventions, based on CRE detection thresholds and compliance levels. Results highlight the benefits of coordinated regional approaches in reducing CRE prevalence and transmission, emphasizing the importance of early and collective action across healthcare facilities. |
| 14 | Lin et al. | 2021 | The model is a hierarchical metapopulation framework that simulates patient movement and CRE (carbapenem-resistant Enterobacteriaceae) transmission across Maryland, encompassing acute-care hospitals (ACHs), long-term care facilities (LTCFs), skilled nursing facilities (SNFs), and communities. It tracks patients through four health states—susceptible, at-risk due to antibiotics, colonized, and infected—with transitions informed by patient flow data and biological parameters. The model evaluates interventions such as selective ICU screening, statewide electronic registries, and predictive algorithms to assess their cost-effectiveness and impact on CRE colonization and infection rates. Results highlight that a statewide registry reduces CRE infections and is significantly more cost-effective than individual hospital-level surveillance measures. |
| 15 | Toth et al. | 2017 | The model is an agent-based simulation of a regional network of healthcare facilities, including a long-term acute care hospital (LTACH), three acute-care hospitals (ACHs), and six nursing homes (NHs). It tracks patient movement and CRE transmission, incorporating facility-specific dynamics such as length of stay, transfer patterns, and transmission rates. The model evaluates the impact of LTACH-focused interventions, such as active surveillance and enhanced isolation, on regional CRE prevalence and transmission. Results highlight the efficiency of targeting LTACHs for interventions, demonstrating significant reductions in regional CRE transmission and prevalence, particularly when implemented early in an outbreak. |
| 16 | Knight et al. | 2018 | The model is a compartmental, deterministic framework designed to quantify antibiotic-resistant bacteria (ARB) acquisition in hospitals and communities. It simulates the flow of individuals between hospital and community settings, tracking antibiotic use, ARB transmission, and acquisition dynamics. Key factors include rates of antibiotic exposure, transmission, resistance clearance, and hospital stay durations. The model demonstrates that most ARB acquisition occurs in the community, even when ARB burden in hospitals is significant, suggesting targeted interventions in community settings could be more impactful in reducing overall ARB levels. |
| 17 | MacFadden et al. | 2019 | The model is a deterministic, compartmental framework designed to simulate the transmission of extended-spectrum beta-lactamase-producing *Escherichia coli* (ESBL-E. coli) within and between hospital and community settings. It incorporates multiple states of colonization (resistant, susceptible, or uncolonized) and stratifies patients by hospital length of stay and antibiotic usage. The model evaluates the relative impact of community versus hospital antibiotic stewardship interventions on ESBL-E. coli prevalence, highlighting the dominant role of community antibiotic use in driving resistance in both settings. Results underscore the interconnectedness of community and hospital dynamics and the need for integrated stewardship approaches. |
| 18 | Talaminos et al. | 2016 | The model is a modified SEIS (Susceptible–Exposed–Infected–Susceptible) compartmental framework designed to study the epidemiology of ST131 Escherichia coli across community, hospital, and long-term care settings. It incorporates deterministic and probabilistic quantification of population flows and evaluates the effects of interventions like reducing person-to-person transmission and antimicrobial exposure. The model demonstrates that sustained reductions in transmission have a significant impact on lowering infection rates, particularly for non-ESBL-producing isolates, while reducing antimicrobial exposure alone has limited effects. These insights highlight the importance of targeting transmission pathways for effective control strategies. |
| 19 | Godijk et al. | 2022 | The model is a continuous-time deterministic framework analyzing the dynamics of plasmid-based beta-lactam resistance in Escherichia coli across hospital, recently hospitalized, and community populations. It examines whether resistance mechanisms result in "replacement" (where resistant infections substitute susceptible ones without increasing total infections) or "addition" (where resistance increases overall infection rates). The model incorporates fitness costs, benefits like increased transmission, decreased clearance, plasmid transfer, and antibiotic selection pressure, assessing their impact on colonization and infection prevalence. Findings suggest that only increased virulence can elevate the total number of infections, while other mechanisms primarily drive replacement. |
| 20 | Haverkate et al. | 2017 | The study utilizes a mathematical household transmission model and a deterministic population model to quantify the spread of extended-spectrum beta-lactamase (ESBL)-producing bacteria within and between households. The household model evaluates person-to-person transmission, background acquisition, and decolonization rates, while the population model incorporates household dynamics, hospital acquisition, travel, and external sources to estimate community-level ESBL prevalence. Results highlight significant within-household transmission and suggest that community prevalence requires contributions from external sources beyond hospital and household transmission alone. This integrated approach underscores the complexity of controlling ESBL spread in community settings. |
| 21 | Salazar-Vizcaya et al. | 2022 | The model is a deterministic framework simulating the spread of ESBL-producing Klebsiella pneumoniae in hospitals and communities, integrating data on antimicrobial consumption and resistance over 13 years in a Swiss region. It quantifies the impact of public health interventions such as reductions in antimicrobial use, in-hospital transmission, and external colonization sources. The model highlights the dominant influence of overall antimicrobial consumption on prevalence, with external colonization contributing significantly to observed rates. Projections suggest reducing antimicrobial consumption would have the greatest effect on controlling future prevalence. |
| 22 | Bartsch et al. | 2021 | The model is an agent-based simulation using the Regional Healthcare Ecosystem Analyst (RHEA) to study the spread of multidrug-resistant organisms (MDROs) such as MRSA and CRE across 102 healthcare facilities in Orange County, California. It tracks patient movements between hospitals, long-term acute care hospitals, and nursing homes, simulating interventions like improved contact precautions, interfacility communication, and decolonization strategies. The model evaluates the effectiveness of these interventions in reducing MDRO prevalence and transmission over three years, highlighting decolonization as the most impactful approach. Results demonstrate the utility of computational modeling in optimizing regional infection control strategies. |
| 23 | Belik et al. | 2016 | The model is an agent-based simulation designed to study the spread of antibiotic-resistant pathogens within a network of 176 hospitals in a German federal state. Using anonymized patient referral data, the model represents a susceptible-infected-susceptible (SIS) epidemic process, simulating patient movements and pathogen transmission across hospitals and the community. It evaluates the impact of control measures, particularly patient screening and isolation, on reducing endemic prevalence. Results indicate that screening approximately 30–40% of incoming patients can halve the prevalence within 100 days, providing a framework for assessing epidemic control strategies in hospital networks. |
| 24 | Van Den Dool et al. | 2016 | The model is a stochastic network-based framework that simulates the spread of antimicrobial-resistant pathogens across a healthcare network, including hospitals and nursing homes in the Netherlands. It uses a susceptible-infected-susceptible (SIS) structure to model transmission within institutions and patient transfers between them. Results show that nursing homes, due to longer patient stays, can significantly sustain and amplify pathogen spread, even when in-hospital transmission is low. This highlights the importance of including nursing homes in regional and national infection control strategies to prevent widespread outbreaks. |
| 25 | Van Kleef et al. | 2017 | The model is a deterministic compartmental framework designed to study the transmission dynamics of antibiotic-resistant and antibiotic-sensitive bacterial strains across hospital and community settings. It explicitly incorporates patient-to-healthcare worker interactions and the movement of colonized individuals between hospitals and the community. The model evaluates the impact of hand hygiene as a non-specific infection control intervention, highlighting its disproportionate effects on resistant strains due to their adaptation to hospital environments. Results show that while resistant strain prevalence declines significantly, sensitive strain prevalence may remain stable or increase slightly due to reduced competition, illustrating the complex dynamics of infection control interventions. |
| 26 | Di Ruscio et al. | 2019 | The model is a stochastic individual-based simulation designed to examine the transmission dynamics of MRSA within community, hospital, and nursing home settings in Norway, a low-prevalence country. It incorporates detailed sociodemographic structures, patient flows, and infection control measures to simulate colonization, infection, and transmission events. Results reveal that while MRSA transmission is not self-sustaining locally (Re < 1), importations drive an increasing prevalence, with households accounting for about half of all new colonizations. Hospitals remain the primary source of symptomatic infections, emphasizing the importance of strict infection control measures in healthcare to mitigate the impact of growing community prevalence. |
| 27 | Gowler et al. | 2022 | The model analyzes the transmission dynamics of methicillin-resistant *Staphylococcus aureus* (MRSA) in healthcare settings, assessing how assumptions about asymptomatically colonized individuals influence estimates of intervention effectiveness. It evaluates scenarios where colonized individuals have higher hospital admission rates or longer hospital stays than uncolonized individuals, finding that such assumptions can bias results, particularly for interventions targeting hospital-based transmission. The study emphasizes the need for realistic modeling assumptions to improve the validity of healthcare-associated infection intervention simulations. |
| 28 | Rocha et al. | 2020 | The model is a high-resolution data-driven contact network simulation of MRSA spread within a large hospital system in Sweden, based on real patient flow data. It captures the spatiotemporal dynamics of patient interactions across 743,182 individuals and 485 hospitals over 3,059 days. The model highlights heterogeneous contact patterns, identifying super-spreader patients and non-linear epidemic growth due to dynamic network structures. Simulations suggest that targeted strategies, such as admission screening and improved hygiene, are critical for controlling outbreaks in interconnected healthcare systems. |
| 29 | Piotrowska et al. | 2020 | The model is a deterministic framework that integrates a susceptible-infectious-susceptible (SIS) structure to simulate the spread of resistant pathogens in hospital-community networks. It incorporates patient flow dynamics, linking hospitals with associated communities, and allows for inter-hospital transfers. By analyzing reproduction numbers for single hospital stays (RA) and the entire infectious period (R0), the model identifies conditions for persistent colonization and the role of readmissions in sustaining transmission. Simulations reveal that increasing patient transfers dilutes prevalence across the network, lowering system-wide prevalence, while hospitals with high R0 remain critical sources of transmission. This model highlights the importance of targeted interventions, such as screening and readmission management, in controlling healthcare-associated infections. |

1. “**Community” in this context is considered any setting outside short-term acute care hospital (STACH), such as home, dorms, universities, and correctional facilities. Community can also include other types of healthcare settings that are not considered STACH, e.g. long term care facilities, rehab/subacute care facilities, and skilled nursing homes.** **We will also include healthcare workers as part of the community.** **Do not include any studies that ONLY investigate acute care hospital setting.** We define STACH *as acute care hospital, burn care hospital, community hospital, critical access hospital, field hospital, general hospital, geriatric hospital, high volume hospital, hospital ship, low volume hospital, magnet hospital, mental hospital, military hospital; pediatric hospital; private hospital; public hospital; rural hospital; safety net hospital; teaching hospital; university hospital; and trauma care hospital*. [↑](#footnote-ref-2)
